# Virome Identification and Characterization of *Fusarium sacchari* and *F. andiyazi*: Causative Agents of Pokkah Boeng Disease in Sugarcane

**DOI:** 10.3389/fmicb.2020.00240

**Published:** 2020-02-19

**Authors:** Ziting Yao, Chengwu Zou, Na Peng, Yu Zhu, Yixue Bao, Qiujuan Zhou, Qingfa Wu, Baoshan Chen, Muqing Zhang

**Affiliations:** ^1^State Key Lab for Conservation and Utilization of Subtropical Agric-Biological Resources, Guangxi University, Nanning, China; ^2^Guangxi Key Laboratory of Sugarcane Biology, Guangxi University, Nanning, China; ^3^College of Life Sciences and Technology, Guangxi University, Nanning, China; ^4^Hefei National Laboratory for Physical Sciences at the Microscale, University of Science and Technology of China, Hefei, China

**Keywords:** *Fusarium sacchari*, *F. andiyazi*, RNA_sequencing, mycovirus, virus diversity

## Abstract

*Fusarium sacchari* and *Fusarium andiyazi* are two devastating sugarcane pathogens that cause pokkah boeng disease (PBD) in China. RNA_Seq was conducted to identify mycoviruses in *F. sacchari* and *F. andiyazi* isolates collected from PBD symptom-showing sugarcane plants across China. Fifteen isolates with a normal, debilitated, or abnormal phenotype in colony morphology were screened out for the existence of dsRNA from 104 *Fusarium* isolates. By sequencing the mixed pool of dsRNA from these *Fusarium* isolates, a total of 26 contigs representing complete or partial genome sequences of ten mycoviruses and their strains were identified, including one virus belonging to *Hypoviridae*, two mitoviruses with seven strains belonging to *Narnaviridae*, one virus of *Chrysoviridae*, and one alphavirus-like virus. RT-PCR amplification with primers specific to individual mycoviruses revealed that mitoviruses were the most prevalent and the alphavirus-like virus and chrysovirus were the least prevalent. In terms of host preference, more mitoviruses were found in *F. andiyazi* than in *F. sacchari*. Fusarium sacchari hypovirus 1 with a 13.9 kb genome and a defective genome of 12.2 kb, shares 54% identity at the amino acid level to the Wuhan insect virus 14, which is an unclassified hypovirus identified from insect meta-transcriptomics. The alphavirus-like virus, Fusarium sacchari alphavirus-like virus 1 (FsALV1), seemed to hold a distinct status amid fungal alphavirus-like viruses, with the highest identity of 27% at the amino acid level to Sclerotium rolfsii alphavirus-like virus 3 and 29% to a hepevirus, Ferret hepatitis E virus. While six of the seven mitoviruses shared 72–94% identities to known mitoviruses, Fusarium andiyazi mitovirus 2 was most similar to Alternaria brassicicola mitovirus with an identity of only 49% between the two viruses. Transmission of FsALV1 and Fusarium sacchari chrysovirus 1 (FsCV1) from *F. sacharri* to *F. commune* was observed and the characterization of the four-segment dsRNA chrysovirus was performed with aid of electron microscopy and analysis of the encapsidated RNAs. These findings provide insight into the diversity and spectrum of mycoviruses in PBD pathogens and should be useful for exploring agents to control the disease.

## Introduction

Pokkah boeng disease (PBD) is an airborne fungal disease caused by *Fusarium fujikuroi* species complex (FFSC) and is responsible for severe yield losses in susceptible varieties of sugarcane worldwide. The FFSC species *F. sacchari* is associated with both PBD and sugarcane wilting (Viswanathan et al., 2017) as well as fruit rot disease in bananas (Abd Murad et al., 2017), spear rot of oil palm (Suwandi et al., 2018), and even mycotic keratitis among sugarcane farmers (Bansal et al., 2016). The FFSC species *F. andiyazi* has been reported to cause wheat head blight (Wang et al., 2015), ear rot of maize (Bandara et al., 2017), and stalk rot of sorghum (Zhang et al., 2014). Recently, our team found *F. sacchari* and *F. andiyazi* strains with various phenotypes in Guangxi, Fujian, and Yunnan, which are the major sugarcane producing provinces in China (Wang J. et al., 2018; Meng et al., 2020). As one of the most important fungal diseases of sugarcane, PBD has long impacted sugarcane production and most sugarcane varieties grown in China are susceptible (Lin et al., 2014). Although the use of antifungal agents has been somewhat effective in controlling PBD, FFSC readily develops resistance to these treatments (Xu et al., 2019). Mycoviruses used as biocontrol agents could be an alternative approach to reduce the economic impact of *F. sacchari* and *F. andiyazi* on sugarcane crops.

The rate of mycovirus discovery was accelerated by next-generation sequencing (NGS), which allows a comprehensive analysis of fungal meta-transcriptomes and virus-derived small RNA (vsiRNA) populations. Application of these methods revealed the existence of multiple mycoviruses that could be grouped into *Mononegavirales*, *Bunyaviridae*, *Aspiviridae*, *Ourmiavirus*, *Virgaviridae*, *Tymoviridae*, *Tombusviridae*, *Barnaviridae*, *Benyviridae*, *Chrysoviridae*, *Megabirnaviridae*, *Quadriviridae*, *Mymonaviridae*, *Endornaviridae*, *Gammaflexiviridae*, *Genomoviridae*, *Hypoviridae*, *Narnaviridae*, *Partitiviridae*, or *Totiviridae* virus families, although many novel mycoviruses cannot be classified (Vainio et al., 2015; Marzano et al., 2016; Nerva et al., 2016; Donaire and Ayllon, 2017; Mu et al., 2017; Zhu et al., 2018; Velasco et al., 2019). Virome sequencing can be used to characterize potential mycoviruses for bio-control of fungal diseases in plants. Some mycoviruses significantly impact host growth, development, and reproduction, thereby affecting host virulence. Cryphonectria parasitica hypovirus 1 (CHV1) reduces its host parasitic growth and has been successfully used to control chestnut blight (Rigling and Prospero, 2018). Alternaria alternata hypovirus 1 (AaHV1) showed an ability to not only reduce *A. alternate* virulence for leaf spot diseases, but also to confer hypovirulence in *Botryosphaeria dothidea*, which is the pathogen of apple white rot disease (Li et al., 2019). Rhizoctonia solani endornavirus 1 (RsEV1) confers hypovirulence in rice sheath blight fungus *Rhizoctonia solani* (Zheng et al., 2019). The first reported fungal DNA virus, Sclerotinia sclerotiorum hypovirulence associated DNA virus 1 (SsHADV-1), confers hypovirulence to *Sclerotinia sclerotiorum* (Yu et al., 2013). Such biocontrol applications of mycoviruses indicate the value of the diversity of fungal virus pathogens using high throughput virome sequencing.

Mycoviruses that infect *Fusarium* spp. have been identified, including double-stranded RNA (dsRNA) viruses, positive-sense single-stranded RNA (+) (ssRNA) viruses, and negative-sense single-stranded RNA (−) (ssRNA) viruses (Sharma et al., 2018; Wang L. et al., 2018). These mycoviruses infect *F. graminearum*, *F. poae*, *F. circinatum*, *F. asiaticum*, *F. solani*, *F. virguliforme*, *F. incarnatum*, *F. langsethiae*, *F. coeruleum*, *F. globosum*, *F. boothii*, and *F. oxysporum*, but there are no reports on mycoviruses that infect *F. sacchari* or *F. andiyazi*. Some mycoviruses are associated with hypovirulence, such as Fusarium graminearum mycovirus-China 9 (FgV-ch9) and Fusarium graminearum hypovirus 2 isolate FgHV2/JS16, which can reduce the virulence of *F. graminearum*. F. graminearum mycoviruses can be easily transmitted to other isolates using protoplast or hyphal fusion techniques (Chu et al., 2002; Darissa et al., 2011; Li et al., 2015). The discovery of hypovirulence-associated viruses that can overcome transmission barriers suggests that mycoviruses might be able to use for control of *Fusarium* fungal diseases.

RNA sequencing techniques have been used for the efficient discovery of novel viruses in fungi, including *Benyviridae*, *Ophioviridae*, and *Virgaviridae* (Marzano et al., 2016), as well as 10 viruses in five *S. sclerotiorum* strains (Khalifa et al., 2016), 17 mycoviruses in a *F. poae* strain (Osaki et al., 2016), and a large number of mycoviruses in *Rhizoctonia solani* AG2-2 LP isolates (Picarelli et al., 2019). In this study, we identified and characterized mycoviruses in the isolates of *F. sacchari* and *F. andiyazi* through RNA_Seq analysis and RT-PCR amplification.

## Results

### Metatranscriptomic Identification of Mycoviruses in *F. sacchari* and *F. andiyazi*

**FIGURE 1:**
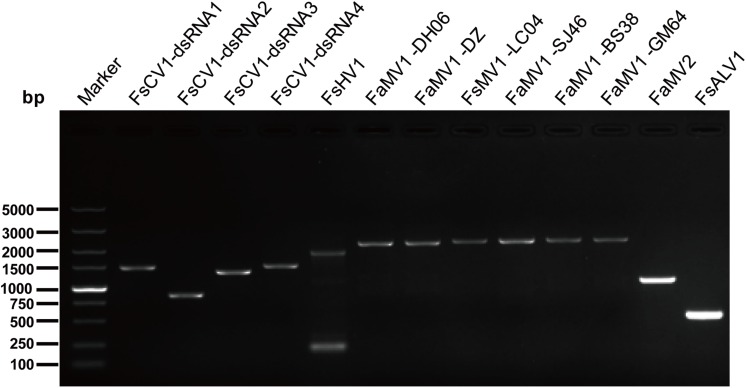
RT-PCR confirmation of mycovirus contigs. RT-PCR confirmation of *de novo* assembled mycovirus contigs from *Fusarium sacchari* and *F. andiyazi* isolates generated by Illumina sequencing. The primers were designed according to the contigs’ sequences (genomic sequences of putative mycoviruses). Primers pairs used and predicted sizes of amplicons are listed in Supplementary Table S2. Lane M, DNA marker, 2,000 bp DNA Ladder (Takara Bio Inc., Japan); Lane 1 to 11, abbreviate of viruses (see Table 1 for detail), Lane H_2_O, ddH_2_O was instead of control RT products.

The gel electrophoresis of dsRNA revealed that nine *F. andiyazi* and six *F. sacchari* from the 104 *Fusarium* isolates (42 *F. sacchari*, 41 *F. andiyazi* and 21 *F. proliferatum*) tested positive for dsRNA presence (Supplementary Figure S1). These dsRNAs were used for complete genome sequencing. After removal of low-quality and host reads, 42,947,006 and 27,056,546 reads from 67,969,464 and 79,658,766 clean reads (paired-end) were *de novo* assembled to produce 35,318 and 41,638 contigs for *F. sacchari* and *F. andiyazi*, respectively. Twenty-six contigs, representing partial or complete mycovirus genome segments, were obtained by BLAST alignment against a GenBank nt database (Table 1). RT-PCR amplification further confirmed that these putative viruses indeed existed in the fungal isolates (Figure 1). Of the ten putative mycoviruses, nine were predicted to have (+) ssRNA genomes and the remaining one had a dsRNA genome. In the nine (+) ssRNA viruses isolated from the 15 fungal strains, seven were mitoviruses (2,145–2,463 nt), one (13,969 nt) was related to a hypovirus, and another (7,685 nt) was an alphavirus-like virus. The dsRNA virus had four segments (3,518, 2,796, 2,779, and 2,569 nt, respectively) and was most similar to chrysovirus in *Chrysoviridae*.

**TABLE 1:** Assembled sequences with similarity to previously described viruses.

**Number**	**Contig number**	**Contig length**	**Name of putative viruses**	**Virus length (nt)**	**GenBank accession numbers**	**Fungal hosts**	**Best match**	**aa identity (%)**	**Query coverage (%)**	**Genome type**	**Family**	**References**
1	Contig 17922_seq1 Contig 10311 _seq1	13,969 13,959	Fusarium sacchari hypovirus 1 (FsHV1)	13,969	MN295969	*Fusarium sacchari*	Wuhan insect virus 14 (WIV14, YP_009342443.1)	54	86	+ ssRNA	*Hypoviridae*	Shi et al., 2016
	Contig 17922 _seq2	12,289	Fusarium sacchari hypovirus 1-Defective RNA (FsHV1-D RNA)	12,289		*Fusarium sacchari*	Wuhan insect virus 14 (WIV14, YP_009342443.1)	54	89	+ ssRNA	*Hypoviridae*	Shi et al., 2016
2	Contig 3638 _seq1	7,685	Fusarium sacchari alphavirus-like virus 1 (FsALV1)	7,711	MN295968	*Fusarium sacchari*	Sclerotium rolfsii alphavirus-like virus 3 (SraLV3, AZF86095.1)	27	11	+ ssRNA	Unclassified	Zhu et al., 2018
3	Contig 17935_seq1 Contig 9916_seq1 Contig 10052_seq1 Contig 10052_seq2	363 1,128 337 2,463	Fusarium andiyazi mitovirus 1-DH06 (FaMV1)	2,456	MN295970	*Fusarium andiyazi*	Fusarium circinatum mitovirus 2-1 (FcMV2-1, AHI43534.1)	94	100	+ ssRNA	*Narnaviridae*	Martínez-Álvarez et al., 2014
4	Contig 10182_seq1	397	Fusarium andiyazi mitovirus 1-DZ (FaMV1)	2,145	MN295971	*Fusarium andiyazi*	Fusarium circinatum mitovirus 2-1 (FcMV2-1, AHI43534.1)	92	100	+ ssRNA	*Narnaviridae*	Martínez-Álvarez et al., 2014
5	Contig 17543_seq3	2,371	Fusarium sacchari mitovirus 1-LC04 (FsMV1)	2,371	MN295976	*Fusarium sacchari*	Fusarium circinatum mitovirus 2-1 (FcMV2-1, AHI43534.1)	72	99	+ ssRNA	*Narnaviridae*	Martínez-Álvarez et al., 2014
6	Contig 8314_seq1 Contig 6632_seq1 Contig 9435_seq2	309 208 913	Fusarium andiyazi mitovirus 1-SJ46 (FaMV1)	2,302	MN295972	*Fusarium andiyazi*	Fusarium circinatum mitovirus 2-1 (FcMV2-1, AHI43534.1)	72	100	+ ssRNA	*Narnaviridae*	Martínez-Álvarez et al., 2014
7	Contig 6109_seq2 Contig 17943_seq1 Contig 17938_seq1	350 232 243	Fusarium andiyazi mitovirus 1-BS38 (FaMV1)	2,184	MN295973	*Fusarium andiyazi*	Fusarium circinatum mitovirus 2-1 (FcMV2-1, AHI43534.1)	73	100	+ ssRNA	*Narnaviridae*	Martínez-Álvarez et al., 2014
8	Contig 9435_seq1	262	Fusarium andiyazi mitovirus 1-GM64 (FaMV1)	2,178	MN295974	*Fusarium andiyazi*	Fusarium circinatum mitovirus 2-1 (FcMV2-1, AHI43534.1)	73	100	+ ssRNA	*Narnaviridae*	Martínez-Álvarez et al., 2014
9	Contig 5918_seq1 Contig 182418_seq1 Contig 97776_seq1 Contig 101266_seq1 Contig 165752_seq1	2,372 323 338 260 211	Fusarium andiyazi mitovirus 2 (FaMV2)	2,372	MN295975	*Fusarium andiyazi*	Alternaria brassicicola mitovirus (AbMV, AKN79252.1)	49	90	+ ssRNA	*Narnaviridae*	Unpublished
10	Contig 2971_seq1	3,518	Fusarium sacchari chrysovirus 1, dsRNA1 (FsCV1 dsRNA1)	3,518	MN295964	*Fusarium sacchari*	Fusarium oxysporum f. sp. dianthi mycovirus 1 (FodV1, RdRp, YP_009158913.1)	89	100	dsRNA	*Chrysoviridae*	Lemus-Minor et al., 2015
	Contig 18164_seq1	2,796	Fusarium sacchari chrysovirus 1, dsRNA2 (FsCV1 dsRNA2)	2,796	MN295965		Fusarium oxysporum f. sp. dianthi mycovirus 1 (FodV1, P2, YP_009158914.1)	87	100	dsRNA	*Chrysoviridae*	Lemus-Minor et al., 2015
	Contig 17958_seq1	2,779	Fusarium sacchari chrysovirus 1, dsRNA3 (FsCV1 dsRNA3)	2,779	MN295966		Fusarium oxysporum f. sp. dianthi mycovirus 1 (FodV1, CP, YP_009158915.1)	91	99	dsRNA	*Chrysoviridae*	Lemus-Minor et al., 2015
	Contig 3153_seq1	2,569	Fusarium sacchari chrysovirus 1, dsRNA (FsCV1 dsRNA4)	2,569	MN295967		Fusarium oxysporum f. sp. dianthi mycovirus 1 (FodV1, P4, YP_009158916.1)	92	99	dsRNA	*Chrysoviridae*	Lemus-Minor et al., 2015

### One Novel Virus in the Family *Hypoviridae*

**FIGURE 2:**
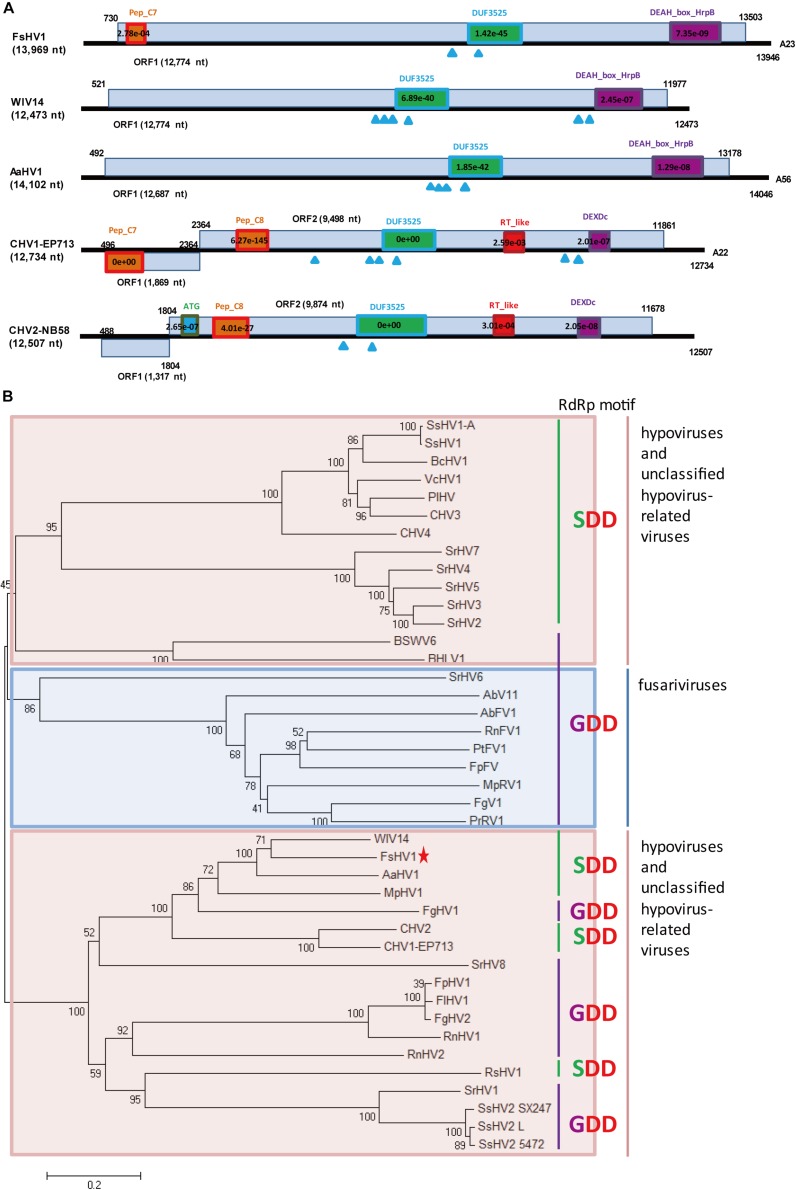
Genomic organization and phylogenetic analysis of FsHV1 with other hypoviruses. **(A)** Genome organization and size of the selected hypoviruses viral sequences. Selected hypoviruses: Fusarium sacchari hypovirus 1 (FsHV1), Wuhan insect virus 14 (WIV14), Alternaria alternata hypovirus 1 (AaHV1), Cryphonectria hypovirus 1 (CHV1), and Cryphonectria hypovirus 2 (CHV2). The ORFs and conserved domains including their sites and E-values identified by NCBI’s conserved domain search were represented by rectangular boxes. Blue triangles indicate the position of predicted transmembrane domains. **(B)** Phylogenetic analysis of hypoviruses and related viruses based on multiple alignments of sequences containing RdRp and RNA_hel domains. A neighbor-joining phylogenetic tree constructed from this alignment for a distance calculated with a Poisson model and a uniform rate between sites using the program MEGA 6.0. Bootstrap percentages (1000 replicates) are shown. Red shading indicates viruses in genus Hypovirus or unclassified; blue shading indicates unclassified fusariviruses. Green lines indicate an SDD tripeptide in the RdRp motif; purple lines indicate a GDD tripeptide in the RdRp motif. The names (full and abbreviated) of selected viruses as well as GenBank accession numbers are listed in Supplementary Table S3. FsHV1 discovered in this work is marked with a red star.

A predicted hypovirus and its defective RNA were identified for *F. sacchari* FJ-FZ06 and GX-FS01. Contig 17922_seq1 and contig 10311_seq1 overlapped. Contig 17922_seq1 had 13,969 nt and contained one complete ORF that was predicted to encode a putative polyprotein of 4,257 aa. Based on BLASTp analysis, the viral-encoded polyprotein had the highest identity (54.1%, coverage 86%, E-value = 0) with the polyprotein encoded by Wuhan insect virus 14 (WIV14), a hypo-like virus identified from insect meta-transcriptomics (Shi et al., 2016). Additionally, this viral protein has identity (29–48%, coverage 45–81%, E-value = 0) to those of Alternaria alternata hypovirus 1 (AaHV1), CHV1, CHV2, and Fusarium graminearum hypovirus 1 (FgHV1) (Shapira et al., 1991; Hillman et al., 1994; Wang et al., 2013; Shi et al., 2016; Li et al., 2019; Supplementary Figure S2). The viral-encoded polyprotein was searched against the conserved domain in NCBI and exhibited three conserved domains, Peptidase_C7 (pfam01830), DUF3525 (pfam12039), and DEAH_box_HrpB (TIGR01970) with low or moderate E-values, which is similar to FgHV1. WIV14 and AaHV1 proteins (with the highest coverage and identity) also contained DUF3525 and DEAH_box_HrpB but lacked the Peptidase_C7 domain. The polyproteins encoded by ORF B of CHV1 and CHV2 had the Peptidase_C8 domain (Figure 2A). In contrast to the ORF B of CHV1 and CHV2, the RdRp domain was not detected in our strains. However, when the viral-encoded and WIV14 polyproteins were aligned with polyproteins of CHV1 and CHV2, nine RdRp core motifs (Ia–VIII) were identified in the region between the DUF3525 and helicase domains (including DEAH_box_HrpB and DEXDc) (Koonin et al., 1991; Supplementary Figure S2A). Based on this region, the viral sequence was grouped in a clade with WIV14, FgHV1, MpHV1 (Macrophomina phaseolina hypovirus 1), CHV1, and CHV2 from the phylogenetic tree of the members of family *Hypoviridae* (Figure 2B; Yaegashi et al., 2012). Owing to its sequence relatedness to hypo- and hypo-like viruses, this newly identified virus is designated as Fusarium sacchari hypovirus 1 (FsHV1). Contig 17922_seq2 had 12,289 nt and contained one complete ORF encoding a putative polyprotein of 3,697 aa, which had a 54% sequence identity to WIV14. These two protein sequences were identical, except that the longer sequence (contig 17922_seq1) had an additional 560 aa at position 1,108. Due to the emergence of defective viral RNA, as was previously observed from other hypo- or hypo-like viruses (Li et al., 2019), the shorter sequence (contig 17922_seq2) could be a defective RNA that has an internal genome deletion. We named contig 17922_seq2 Fusarium sacchari hypovirus 1-Defective RNA (FsHV1-D RNA) (Table 1). FsHV1 was identified in *F. sacchari* strains FJ-FZ06, GX-FS01 and GX-FS03, whereas its D RNA was only detected in GX-FS01.

When the N-terminal region (145–240 aa region) of the FsHV1 putative polyprotein was aligned with papain-like cysteine protease domain regions of polyproteins from other hypo- and hypo-like viruses, the presence of conserved three cysteine protease core residues (cysteine, histidine, and glycine in Supplementary Figure S2B) indicated that FsHV1 encoded polyproteins processed by cysteine protease (Koonin et al., 1991). FsHV1 in the phylogenetic tree clustered with CHV1, CHV2, WIV14, AaHV1, and FgHV1 (Figure 2B). This group has a conserved SDD tripeptide in RdRP motif VI (see Figure 2B), except for FgHV1, which is GDD.

### One Novel Alphavirus-Like Virus

**FIGURE 3:**
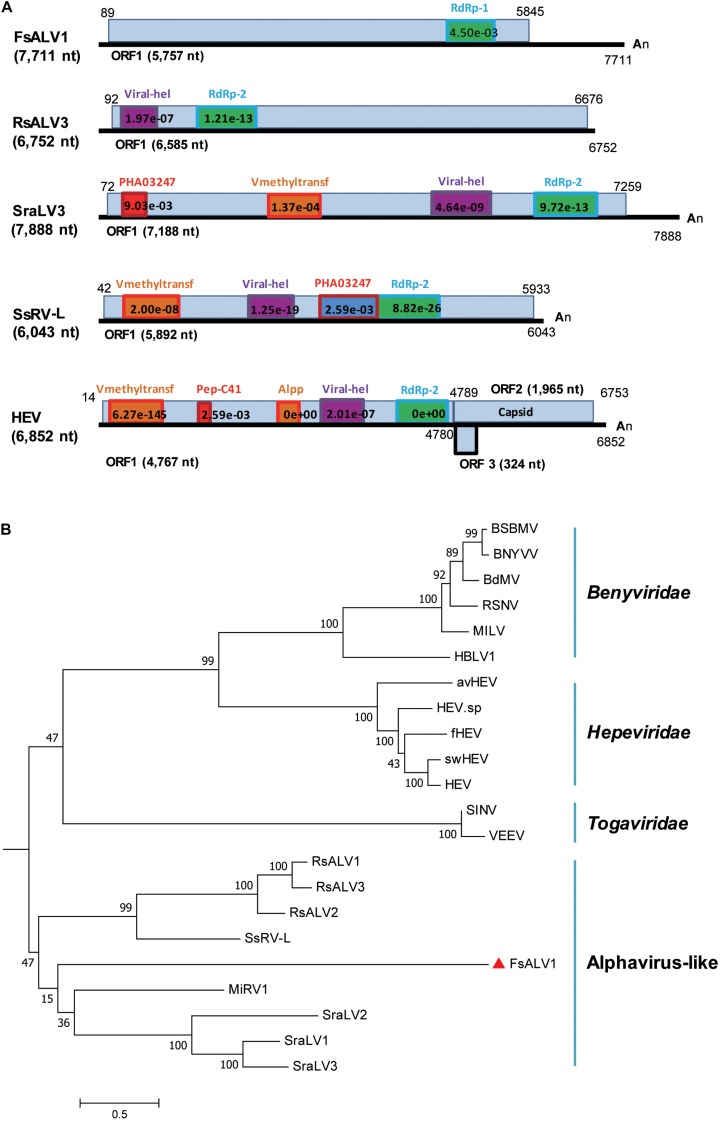
Genomic organization and phylogenetic relationships of FsALV1 with other alphavirus-like viruses and one hepatitis E virus. **(A)** Genome organization and size of the selected viral sequences. Selected viruses: Fusarium sacchari alphavirus-like virus 1 (FsALV1), Rhizoctonia solani alphavirus-like virus 3 (RsALV3), Sclerotium rolfsii alphavirus-like virus 3 (SraLV3), Sclerotinia sclerotiorum RNA virus L (SsRV-L), and hepatitis E virus (HEV). The ORFs and conserved domains including their sites and E-values identified by NCBI’s conserved domain search were represented by rectangular boxes. **(B)** Phylogenetic analysis of alphavirus-like viruses and related viruses based on multiple alignments of sequences containing RdRp domains. A maximum-likelihood phylogenetic tree was generated using MEGA 6.0 with the best-fit model JTT + F + G5 + I. Bootstrap percentages (1000 replicates) are shown. The names (full and abbreviated) of selected viruses as well as GenBank accession numbers are listed in Supplementary Table S3. FsALV1 discovered in this work is marked with a red triangle.

A novel alphavirus-like virus was discovered in *F. sacchari* FJ-FZ04. The 3638_seq1 contig sequence was 7,685 nt in length with a complete ORF encoding a putative protein of 1,920 aa, which had 27% identity to Sclerotium rolfsii alphavirus-like virus 3 (SraLV3) and Ferret hepatitis E virus (fHEV) with 13–24% coverage. Only one incomplete RdRp_1 domain (pfam00680) was present in the viral-encoded polyprotein of our strains, whereas other alphavirus-like viruses encoded the polyprotein with RdRp_2 domain (clo3049) and other domains, including Rhizoctonia solani alphavirus-like virus-1, -2, -3 (RsALV-1, -2, -3), SraLV-1, -2, -3, Sclerotinia sclerotiorum RNA virus L (SsRV-L), and hepeviruses. The polyproteins of SraLV-1, -2, -3, SsRV-L, and hepeviruses had Viral_hel (cl26263) and Vmethyltransf (cl03298) domains, while RsALV3 only possessed the Viral_hel domain (Figure 3A). Homology searches of the RdRp conserved motifs of the above-mentioned viruses indicated eight RdRp core motifs (I–VIII) and a conserved GDD tripeptide present in the region (Supplementary Figure S3). Contig 3638_seq1 grouped with RsALV-1, -2, -3, and MiRV1 from the phylogenetic tree constructed by the RdRp domain, thus forming a distinct clade (Figure 3B). Based on these findings, we named this virus Fusarium sacchari alphavirus-like virus 1 (FsALV1) and detected it in *F. sacchari* FJ-FZ04. We extended the length of FsALV1 to 7,711 nt by RACE, indicating that the initial assembly covered 99.7% of the complete viral genome.

### One Novel and Six Previously Reported Mitoviruses in the Family *Narnaviridae*

**FIGURE 4:**
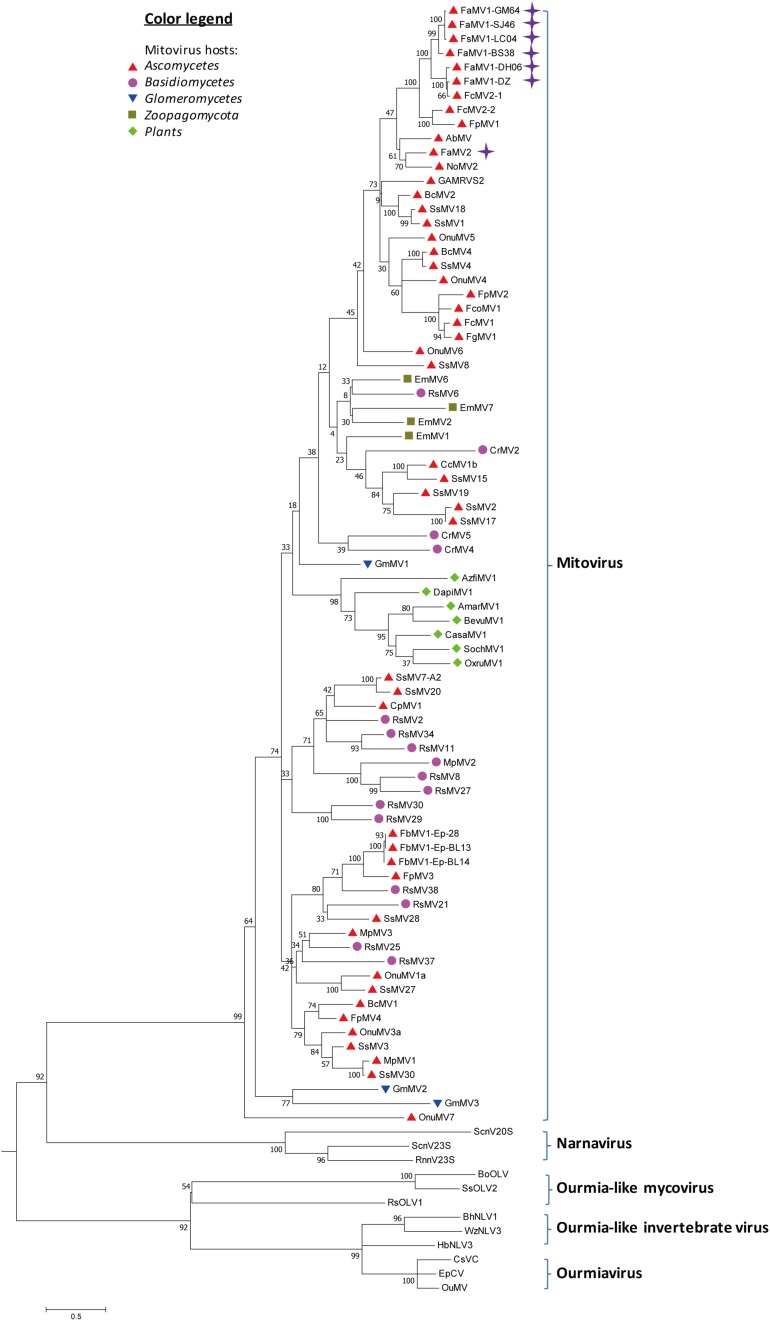
Phylogenetic analysis of Mitoviruses. 91 sequences have been used to produce an alignment starting from viruses belonging to Narnaviruses, Ourmiaviruses and Ourmia-like viruses as outgroup; the phylogenetic tree was built using the neighbor-joining method, the best choice for a distance calculated with a Poisson model and uniform rates between sites using the program MEGA 6.0. Ultrafast bootstrap analysis was performed with 1,000 replicates. Viruses discovered in this work are marked with purple diamonds. Previously identified mitoviruses from *Ascomycota* hosts are labeled with red triangles. Other mitoviruses from other phyla of fungal hosts are labeled: *Basidiomycota*, purple circle; *Glomeromycotina*, blue triangle; *Zoopagomycota*, brown square; plants, green diamond. The names (full and abbreviated) of selected viruses as well as GenBank accession numbers are listed in Supplementary Table S3.

From the RNAseq assembly, we identified 18 sequences encoding for proteins that showed high similarity with viruses from the Narna-levi clade (Shi et al., 2016), but several of them encoded the uncomplete ORFs of Mitovir_RNA_pol (Mitovirus RNA-dependent RNA polymerase; pfam05919). To identify different isolates, we assembled and aligned all of the sequences and designed primers specific for conserved regions, including the complete ORFs. A total of 13 contigs originated from one *F. sacchari* isolate and five *F. andiyazi* isolates were assembled into seven putative mitoviruses (Table 1). These putative mitovirus sequences from *F. sacchari* and *F. andiyazi* were 2.1 to 2.5 kb long, respectively, and encoded the complete Mitovir_RNA_pol domain: two for 708 aa protein, one for 709 aa protein, one for 724 aa protein, one for 727 aa protein, and one for 741 aa protein, suggesting that there might be multiple mitovirus isolates represented by these sequences. The RdRp catalytic motif GDD was searched against the conserved domain database of NCBI (Supplementary Figure S4). BLASTp showed that the top hit was to the Fusarium circinatum mitovirus 2-1 (FcMV2-1) (two contigs, 93 and 94% identity; E-values, 0.0); for the other four contigs, the top hit was the same as FcMV2-1 with lower identity (72∼73% identity; E-values, 0.0); and for the remaining contig, the top hit was to Alternaria brassicicola mitovirus (AbMV) (49% identity; E-value, 0.0). These viral sequences matched the typical pattern for fungal mitoviruses, in which 83% (10/12) of their Trp was encoded by UGA (Supplementary Figure S4). We subsequently included viral sequences encoding the Mitovir_RNA_pol domain in the phylogenetic analysis (Figure 4). All seven sequences clustered in one sub-clade. Six sequences and FcMV2-1 shared 72–94% identity, which were named Fusarium andiyazi mitovirus 1. The remaining sequence shared 49% identity of NoMV2 was named Fusarium andiyazi mitovirus 2 (FaMV2).

### One Strain of a Previously Reported dsRNA Virus in the Family *Chrysoviridae*

**FIGURE 5:**
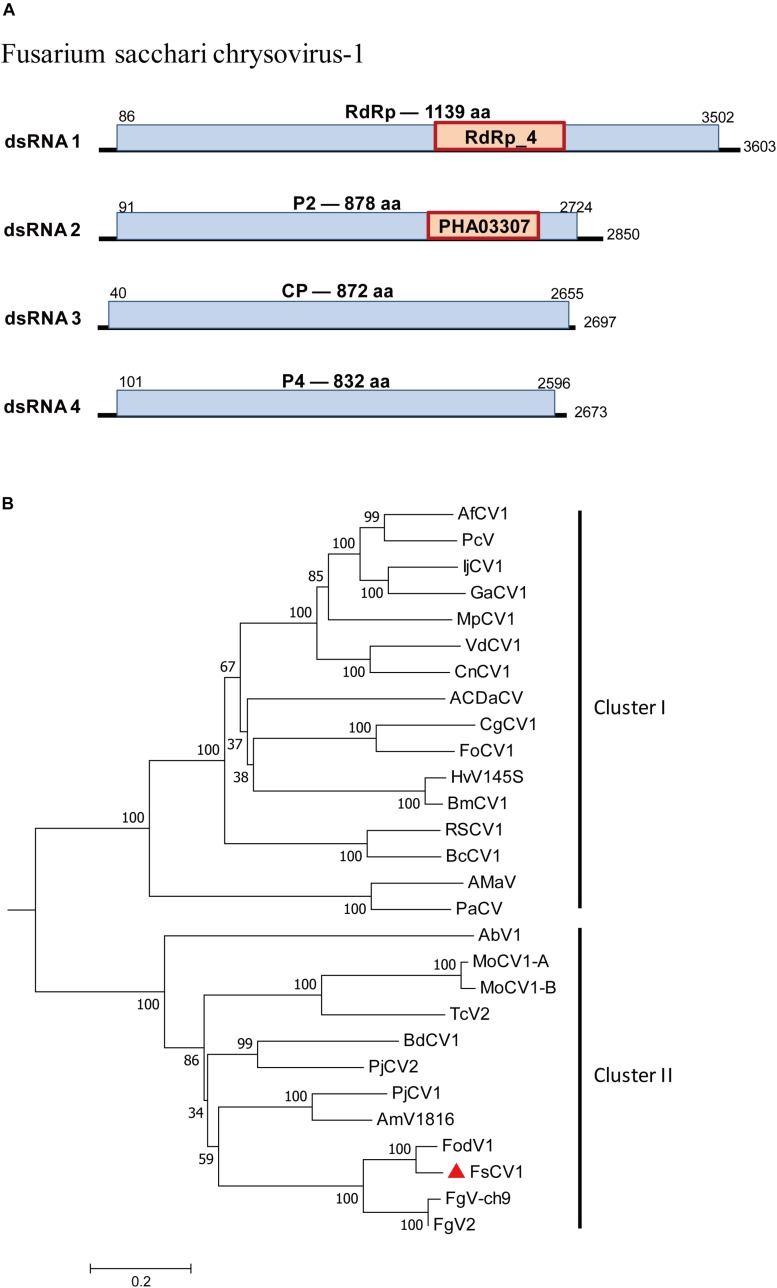
Genomic organization of Fusarium sacchari chrysovirus 1 (FsCV1) and phylogenetic analysis of chrysoviruses and related, unclassified viruses in Family *Chrysoviridae* based on viral RdRp amino acid sequences. **(A)** The FsCV1 genome consists of four dsRNA segments. The ORFs and conserved domains were represented by rectangular boxes. **(B)** A neighbor-joining phylogenetic tree constructed from this alignment for a distance calculated with a Poisson model and a gamma distribution of five rates between sites using the program MEGA 6.0. Bootstrap percentages (1000 replicates) are shown. The names (full and abbreviated) of selected viruses as well as GenBank accession numbers are listed in Supplementary Table S3. FsCV1 discovered in this work is marked with a red triangle.

*Chrysoviridae* is a family of dsRNA viruses with genomes ranging from 11.5 to 12.8 kb that typically include four encapsidated segments at sizes from 2.5 kb to 3.6 kb (Kotta-Loizou et al., 2020). A chrysovirus was detected in *F. sacchari* FJ-FZ04. Four contigs were associated with Fusarium oxysporum f. sp. dianthi mycovirus-1 (FodV1) (Table 1). Contig 2971_seq1 having 3,518 nt contained a complete ORF encoding a putative protein of 1,139 aa that had the highest similarity to the FodV1 RdRp (89% identity). Contig 18164_seq1 consisted of 2,796 nt and contained a complete ORF encoding a putative 878 aa protein, which was similar to the hypothetical protein P2 of FodV1 (87% identity). Contig 17958_seq1 consisted of 2,779 nt and contained a complete ORF encoding a putative 852 aa protein that had the highest similarity to the FodV1 coat protein (91% identity; Table 1). Contig 3153_seq1 consisted of 2,569 nt and contained one complete ORF predicted to encode an 830 aa protein. Blastp analysis showed that this putative protein had sequence similarity to the hypothetical FodV1 protein P4 (92% identity). These four contigs represented a new FodV1 isolate from *F. sacchari* FJ-FZ04. Therefore, we named this sequence Fusarium sacchari chrysovirus 1 (FsCV1), which was closely related to FodV1 based on the phylogenetic tree constructed from the conserved RdRp domains of FodV1 and other selected chrysoviruses using a maximum-likelihood method (Figure 5). FsCV1 shared a similar genomic organization and size as FodV1. *Chrysoviridae* family members could be divided into two distinct clusters from the phylogenetic tree (Zhai et al., 2018). Cluster I contained members of the genus Chrysovirus and 3-segmented chrysovirus-related unclassified viruses. FsCV1 grouped with FodV1 to form a separated evolutionary clade in cluster II, which contained chrysovirus-related, unclassified viruses with four to seven genomic segments (Figure 5).

### Horizontal Transmission of FsALV1 and FsCV1 via Hyphal Fusion

The strain *F. sacchari* FJ-FZ04 was coinfected with FsALV1 and FsCV1, which were confirmed by RT-PCR detection (Figure 1). Virus particles of FsCV1 were purified from the mycelia of FJ-FZ04 using CsCl gradient centrifugation. Agarose gel electrophoresis of the nucleic acids extracted from the 1.30 – 1.40 g cm^–3^ CsCl gradient fractions showed dsRNA bands of FsCV1 (Supplementary Figure S5B). The purified virus particles were isometric and approximately 50 nm in diameter as observed by transmission electron microscopy (Supplementary Figure S5A). RT-PCR amplification of dsRNA extracted from purified virions identified four dsRNA segments (Supplementary Figure S5C).

**FIGURE 6:**
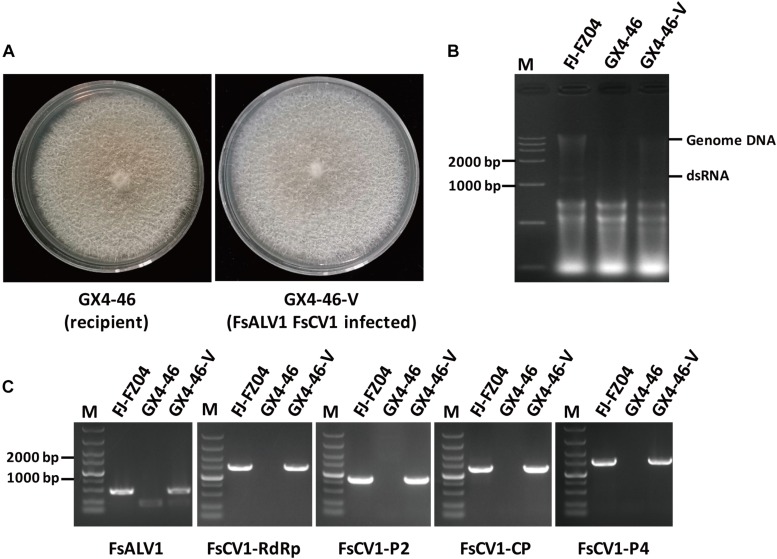
Transmission of FsALV1 and FsCV1 from the donor isolate FJ-FZ04 to the virus-free recipient isolate GX4-46. **(A)** Colony morphology of the virus-free GX4-46 and a virus-infected GX4-46-V after hyphal fusion and selection. Each strain was incubated for 10 days at 25°C on a PDA plate. **(B)** Gel electrophoresis of total Nucleotide from FJ-FZ04 (lane 2), the GX4-46 (lane 3), and virus-infected GX4-46-V after hyphal fusion and selection (lanes 4). Electrophoresis was performed in 1% (w/v) agarose gels. **(C)** Detection of the FsALV1 and FsCV1 dsRNA 1 ∼4 by RT-PCR using specific primers (see Supplementary Table S2). Gel electrophoresis of total Nucleotide from FJ-FZ04 (lane 2), the GX4-46 (lane 3), and virus-infected GX4-46-V after hyphal fusion and selection (lane 4). Electrophoresis was performed in 1% (w/v) agarose gels.

Transmission of the virus from the *F. sacchari* strain FJ-FZ04 which harbored FsCV1 and FsALV1 to the virus-free recipient *F. commune* strain GX4-46, pathogen of sugarcane root rot (Wang J. et al., 2018), were conducted by pairing both strains on PDA plates for a week. After three consecutive subcultures of the recipient strain (designated as *F. commune* GX4-4-V), RT-PCR was performed with primers specific to the FsALV1 and FsCV1. PCR products of the expected sizes were amplified, demonstrating that both FsALV1 and FsCV1 were transmitted from FJ-FZ04 to GX4-46 *via* anastomosis. To exclude the possibility of contamination from the donor strain, the newly obtained virus-harboring *F. commune* GX4-4-V was subjected to re-identification by sequencing the *TEF1*-α gene which was amplified with primer pairs EF1/EF2. The result showed that *TEF1*-α from *F. commune* GX4-4-V was identical to that of the *F. commune* GX4-46 and shared 88.9% identity with the donor strain *F. sacchari* FJ-FZ04. Compared with the virus-free *F. commune* GX4-46, *F. commune* GX4-4-V grew slightly faster on PDA plate, but the pigmentation was slightly reduced (Figure 6).

## Discussion

A complex virome and its diverse viral segments were identified and characterized from 15 isolates of *F. sacchari* and *F. andiyazi* using high-throughput transcriptome sequencing. Some of these detected viruses were predicted to belong to the viral species within the families *Hypoviridae*, *Narnaviridae*, and *Chrysoviridae*, whereas one was an unclassified alphavirus-like (+) ssRNA virus. To the best of our knowledge, this is the first report of a comprehensive analysis on the viral diversity in the isolates of *F. sacchari* and *F. andiyazi*. Among six *F. sacchari* isolates, one hypovirus and one novel alphavirus-like virus as well as one characterized chrysovirus and one mitovirus were detected. Another six characterized mitoviruses and one novel mitovirus were present among nine *F. andiyazi* isolates.

The hypoviruses are known to be associated with hypovirulence and other symptoms, including slow growth, reduced conidiation, or toxin inhibition, in *C. parasitica*, *S. sclerotiorum*, and *F. graminearum* (Li et al., 2015; Marzano et al., 2016; Rigling and Prospero, 2018). The hypoviruses were clustered into two clades based on the RdRp and Hel domains of the polyprotein regions in the recent ICTV report (Suzuki et al., 2018). In this study, FsHV1 clustered with CHV1 and CHV2 in the “alphahypovirus” group. The Peptidase_C7 was found in the 5′ terminus of the FsHV1 encoding polyprotein as well as the 5′ terminus of ORFA of CHV1. The product of the Peptidase_C7 domain in CHV1 is p29 protein, which is a protease acting as a virus-encoded determinant that can alter fungal host phenotypes in CHV1 isolates (Xiong et al., 2019). The p29 protein was able to suppress both virus-induced and agroinfiltration-induced RNA silencing and systemic spread of silencing in GFP-expressing transgenic *Nicotiana benthamiana* line 16c plants (Segers et al., 2006). In addition, p29 has been shown to stimulate membrane and vesicle proliferation by directing p29 to the *trans*-Golgi network (TGN) vesicles through cofractionation with TGN membranes (Jacob-Wilk et al., 2006). ORF B was associated with virus transmission and host hypovirulence by affecting the transmission efficiency of conidiospores and viral accumulation (Lin et al., 2007). Although FJ-FZ06 caused lesions in sugarcane stalks, its virulence awaited certification after the construction of virus-free strains.

Both complete and defective viral genomes of FsHV1 were detected in GX-FS09. For defective FsHV1, no conserved domain was found in the absent region between genome coordinates 4,058 and 5,738. D-RNAs were also detected in CHV1, AaHV1, and other family members, such as Rosellinia necatrix partitivirus 2 (RnPV2). RnPV2 D-RNAs affect parental virus replication and mitigate viral symptoms in a Dicer-like 2 knockout mutant of *C. parasitica* (chestnut blight fungus), which is an artificial host for RnPV2 (Chiba et al., 2016). The discovery of D-RNAs in *F. sacchari* is very important, although further research is required to clarify whether FsHV1-S D-RNA affects viral replication and the fungal host phenotype. The availability of two fully assembled FsHV1 genomes allows for comparison with other members of the family *Hypoviridae*.

Mitoviruses have small plus-strand RNA genomes and are present in various species of fungi. RdRps from FaMV1-DH06 and -DZ were related to FcMV2-1 with 91–92% sequence identity, whereas FsMV1-LC04, FaMV1-SJ46, -BS38, and -GM64 were related to FcMV2-1 with 72–73% identity, suggesting that various FcMV2-1 strains were widely distributed in Yunnan, Guangxi, and Hainan Provinces in China. *F. andiyazi* isolates also appeared more frequently in samples from Yunnan. Another novel mitovirus, FaMV2, was also present in Yunnan Province. The observation that mitoviruses transmit vertically together with host propagation could provide some clues as to why *Fusarium* spp. was more divergent in Yunnan Province. Mitoviruses appeared to reduce virulence (hypovirulence) in various plant pathogens, including *C. parasitica*, *Ophiostoma novo-ulmi*, *S. homoeocarpa*, and *S. sclerotiorum* (Polashock and Hillman, 1994; Hong et al., 1998; Deng and Boland, 2006; Xu et al., 2015). The viral transmission and pathogenicity of mitoviruses from *F. andiyazi* and *F. sacchari* will need to be explored in more detail. Fusarium spp. mitoviruses mainly fell into two sub-clades of the phylogenetic tree, suggesting that their interspecific transmission might not occur as easily in nature as other fungi, such as *Rhizoctonia solani* and *Sclerotinia sclerotiorum* (Mu et al., 2017; Picarelli et al., 2019).

The *F. sacchari* strain FJ-FZ04 was found to be infected by two viruses, one chrysovirus and one unclassified alphavirus-like virus. FsCV1 shared the highest sequence identity with the previously reported virus FodV1, which is associated with virulence and other phenotypic traits of the plant pathogenic fungus *F. oxysporum* f. sp. *dianthi*. FsCV1 grouped with FodV1, FgV2, and FgV-ch9 in cluster II in the phylogenetic tree. These three mycoviruses were confirmed to be associated with phenotypic alterations, including hypovirulence. A high level of viral accumulation of FodV1 has been reported to reduce mycelial growth, conidiation, and virulence (Lemus-Minor et al., 2018). These results were comparable with the previous report for FgV-ch9, whereby higher accumulation in *F. graminearum* was linked to a reduction in conidiation and virulence (Darissa et al., 2011). Thus, it may be interesting to investigate whether FsCV1 can induce hypovirulence in *F. sacchari*. FsALV1 was also found in FJ-FZ04, but whether FJ-FZ04 hypovirulence is also associated with FsALV1 or not will require further studies.

In conclusion, various mycoviruses were detected in *F. sacchari* and *F. andiyazi*, the pathogenic fungi that causes pokkah boeng disease in sugarcane in China. Our results confirmed that mycovirus infection of *F. sacchari* and *F. andiyazi* was widespread in leading sugarcane producing regions and that some of these viruses might be associated with the hypovirulence of *Fusarium* spp.

## Materials and Methods

### Growth of *F. sacchari* and *F. andiyazi* Isolates

*Fusarium sacchari* and *F. andiyazi* isolates were recovered from the diseased leaves of sugarcane (see Supplementary Table S1) and grown on potato dextrose agar (PDA) medium at 28°C. Isolates were kept on PDA plates at 4°C for this study.

### Total RNA Extraction and Purification

Each of the 15 isolates was cultured on a PDA plate for 5–7 days. Total RNA was extracted from the one-gram mycelial mass using a Trizol kit (Takara Bio, Inc., Japan) according to the manufacturer’s instructions. Total RNA (1 μg) from each of the 15 isolates was mixed for RNA_Seq analysis.

### RNA Sequencing

Sequencing was carried out by Beijing Novel Bioinformatics Co., Ltd., using an Illumina HiSeq 2500 instrument. Sequencing libraries were constructed from 15 mixed rRNA-depleted total RNA samples using a TruSeqTM RNA Sample Prep Kit (Illumina, RS-122-2001). After discarding low-quality reads, including paired-end reads less than 100 bp, reads with quality scores <20, and reads containing adapter sequences, clean reads were mapped to the genome of Fusarium spp. using bowtie2 v2.1.0. Reads that did not align concordantly were assembled by a metagenomic *de novo* assembly using Trinity (version: Trinity RNAseq-r2013-02-25). Large contigs with lengths ≥200 nt were obtained and subjected to local BLAST against a nucleotide database using BLASTn. A second alignment was performed against viral protein sequences using BLASTx, which was also used to remove false-positive hits against the nr database. To identify highly divergent viruses, contigs with the best match to viral protein or viral nucleotide sequences and E-values <1 × 10^–3^ were retained.

### Confirmation of Putative Mycoviruses

To verify the presence of putative mycovirus in the strains, cDNAs were synthesized using Moloney murine leukemia virus (M-MLV) transcriptase (Takara Bio, Inc., Japan), and viral sequences were detected by RT-PCR using specific primers designed on the 26 assembled contigs that matched viral sequences (Supplementary Table S2). To complete the sequences of the alphavirus-like virus genomes, the 5′- and 3′-terminal sequences were determined using the SMARTer RACE 5′/3′ kit (Clonetech Laboratories, Mountain View, CA. Primers 5′-1R (5′-ATGGGTTTAAGGAGAGAGTGCGAGAGTCTATGTTCTT GAATGTC-3′) and 5′-2R (5′-CGAGCCAAGAGAATAGAGT AGGAGAG-3′) were used for 5′ random amplification of cDNA ends (RACE) as outer and inner primers, respectively. Primers 3′-1F (5′-AAGCGGCACCTAGACCTACGACATTGGCAGACA-3′) and 3′-2F (5′-GTGACGGAGTACGACCAGTCTT-3′) were used for 3′ RACE as outer and inner primers, respectively.

### Phylogenetic Analysis

The highly homologous viral nucleotide sequences or their deduced amino acid sequences of contigs were aligned against known viral nucleic acids and proteins in GenBank. The phylogenetic trees from the viruses and accession numbers of the viral gene(s) (Supplementary Table S3) were constructed using the Maximum likelihood method or Neighbor-Joining method with a bootstrap value of 1,000 replicates through MEGA 7.0^1^.

### Purification of Virus Particles

Frozen mycelia (6 g) were homogenized in 0.1 M sodium phosphate buffer (pH 7.4) with a mixer at a frequency of 25 Hz for 1 min. The suspension was shaken for 30 min at 4°C, mixed with 20% (v/v) of (1:1) chloroform:n-butanol, and centrifuged at 8,000 × *g* for 10 min. The chloroform:n-butanol step was repeated 2–3 times until the red pigment was removed from the supernatant. PEG6000 and NaCl were added to the supernatant to final concentrations of 8% (w/v) and 1% (w/v), respectively. After 1 h of incubation at 4°C, the precipitate was collected by centrifugation (10,000 × *g* at 4°C for 15 min), and the pellet was resuspended in 10 mL of 0.05 M sodium phosphate buffer (pH 7.0) at 4°C for 3 h. The pellet was resuspended in 1 mL of 0.05 M sodium phosphate buffer (pH 7.0) and further centrifuged using CsCl density equilibrium centrifugation (25,000 rpm at 5°C for 22 h). Virus-like particles were stained with 2% (w/v) phosphotungstic acid and examined by transmission electron microscopy (HT-7700).

### Hyphal Fusion

Virus infection via hyphal anastomosis was performed using the virus strain FJ-FZ04 as the donor and the virus-free *Fusarium commune* isolate GX4-46 as the recipient. Mycelial plugs of donor and recipient isolates were placed 2 cm apart on a 9 cm PDA plate and incubated at 28°C for 7 days. Several plugs picked up from the border of strain GX4-46 in the region between the two colonies were sub-cultured three times on PDA plates.

## Data Availability Statement

The datasets generated for this study can be found in GenBank MN295969 MN295968, MN295970, MN295971, MN295976, MN295972, MN295973, MN295974, MN295975, MN295964, MN295965, MN295966, and MN295967.

## Author Contributions

ZY, MZ, and BC conceived and designed the experiments. ZY, CZ, NP, YZ, QZ, and QW performed the experiments. ZY, CZ, YB, BC, and MZ analyzed the data and wrote the manuscript.

## Conflict of Interest

The authors declare that the research was conducted in the absence of any commercial or financial relationships that could be construed as a potential conflict of interest.

## Footnotes

1 http://www.megasoftware.net/megamacBeta.php
