# Supplementary material for: Virome Identification and Characterization of Fusarium sacchari and F. andiyazi: Causative Agents of Pokkah Boeng Disease in Sugarcane
Source: Front Microbiol. 2020 Feb 19;11:240. doi: 10.3389/fmicb.2020.00240 (PMC7042383; doi:10.3389/fmicb.2020.00240)
Supplement: Supplementary file 1 [file Data_Sheet_1.docx]

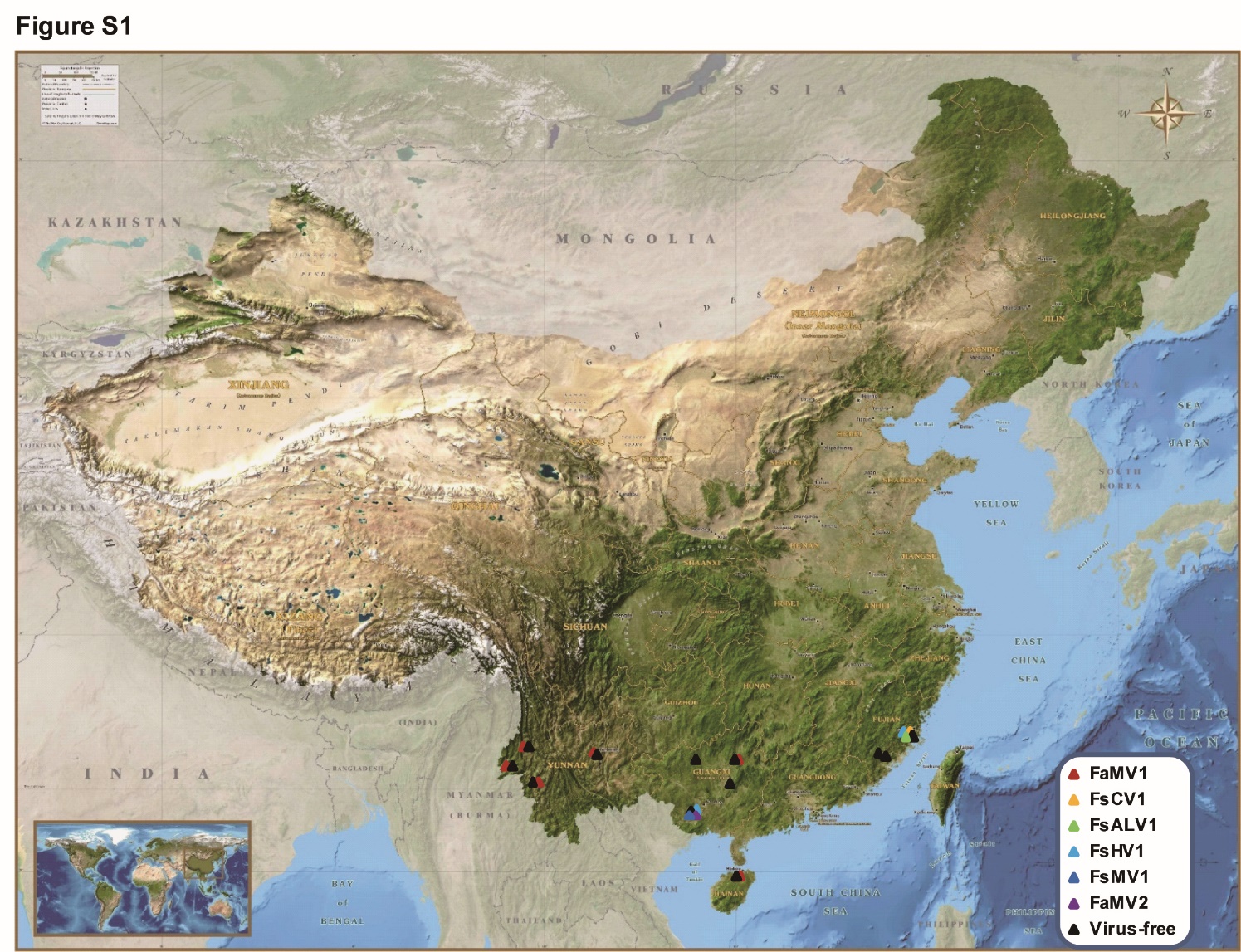


**Figure S1.** Map of sampling site for *Fusarium sacchari* and *F. andiyazi* isolates in China. The types of mycovirus detected in the isolates were represented by triangles.


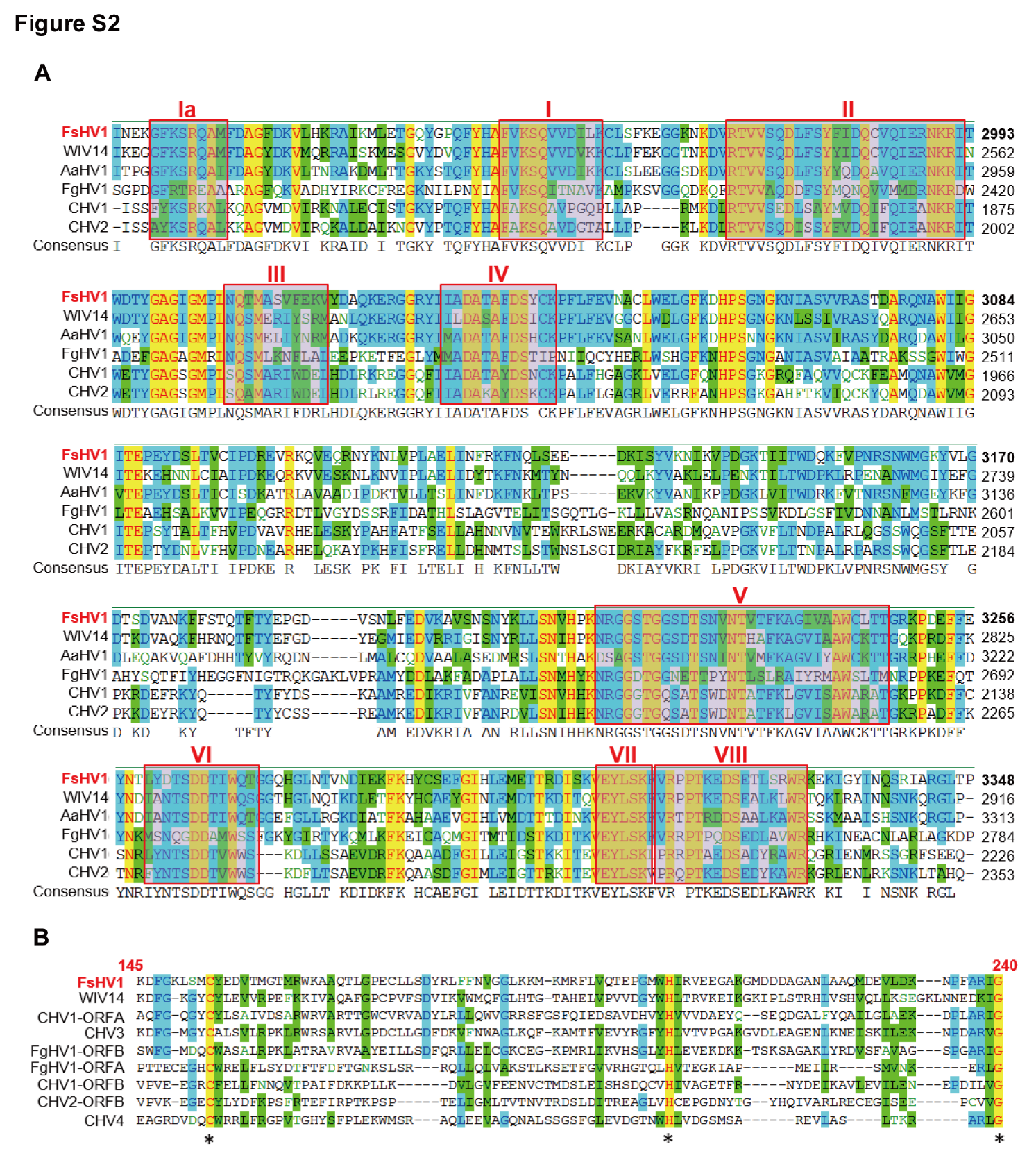


**Figure S2.** Genomic properties of Fusarium sacchari hypovirus 1 (FsHV1). **(A)** Amino acid sequence alignment of the region corresponding to RdRp domain. The position of nine core RdRp motifs were highlighted. Numbers refer to the amino acid position in the ORF. **(B)** Amino acid sequence alignment of the region corresponding to cysteine protease. Conserved three cysteine protease core residues (cysteine (**C**), histidine (**H**), and glycine (**G**)) were labeled with asterisk. Numbers refer to the amino acid position in the FsHV1 ORF.


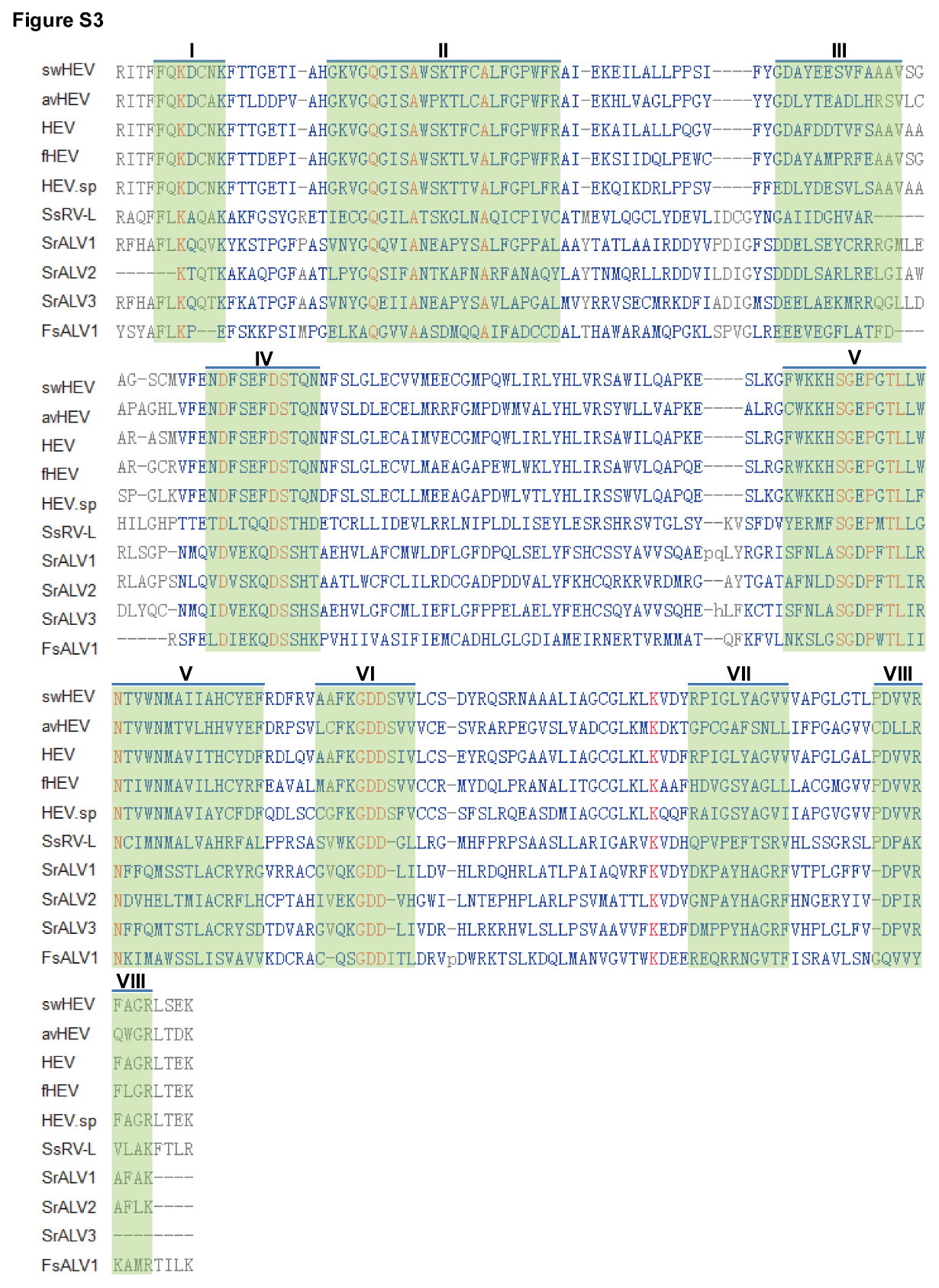


**Figure S3.** Amino acid sequence alignment of the putative RdRp motifs of Fusarium sacchari alphavirus-like virus 1 (FsALV1) and those of selected viruses in the genus Hepevirus and alphavirus-like viruses. The positions of the conserved motifs in these motifs (shaded areas) are indicated with horizontal lines above the shaded areas. Red letters indicate identical amino acid residues, and blue letters indicate similar residues. Numbers refer to the amino acid position in the ORF. See Table S3 for abbreviations of virus names and viral protein accession numbers.


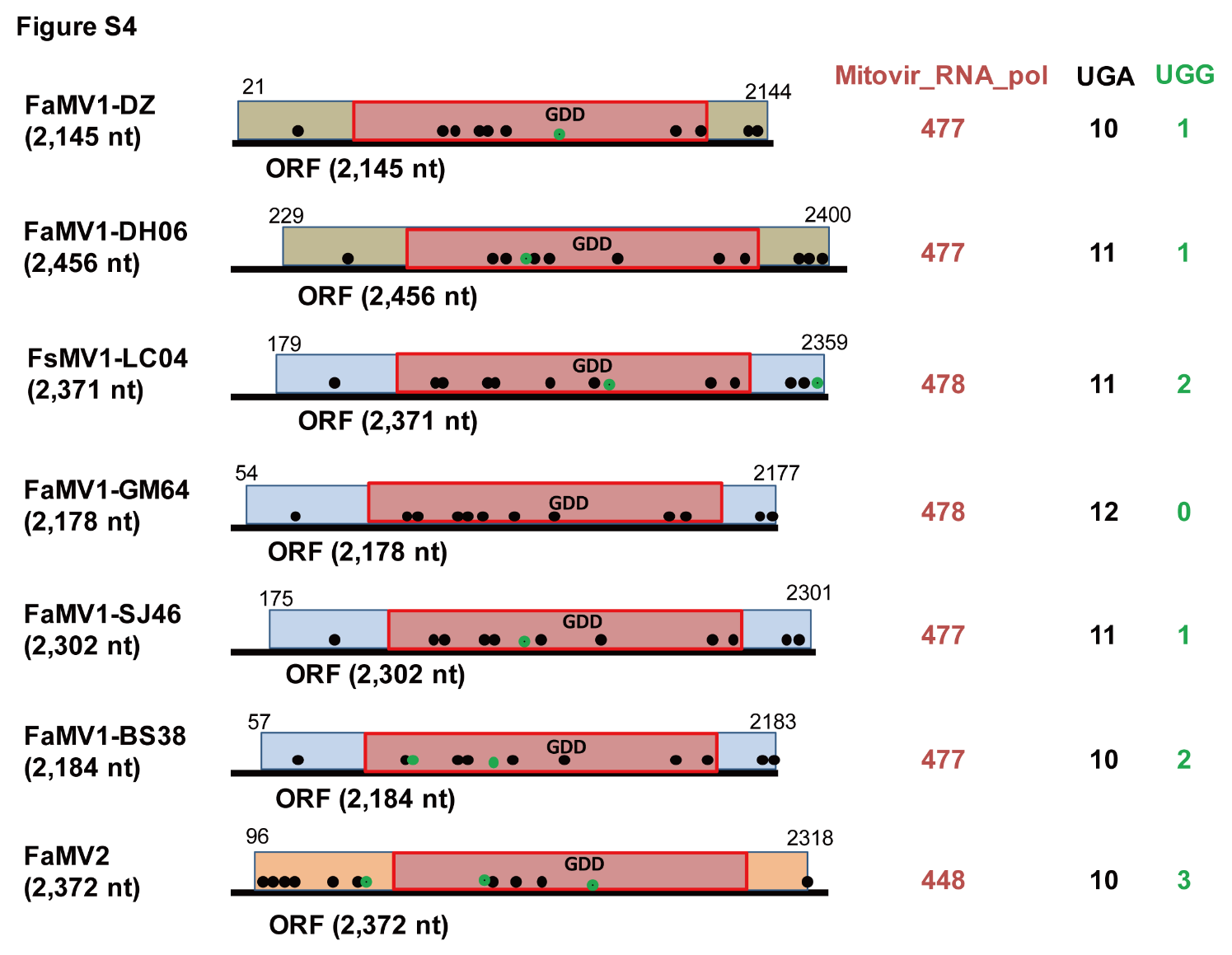


**Figure S4.** Genome organization of Fusarium andiyazi mitoviruse 1 from five *F. andiyazi* isolates (FaMV1- ), Fusarium andiyazi mitoviruse 2 (FaMV2), and Fusarium sacchari mitoviruse 1 from one *Fusarium sacchari* isolate (FsMV1-LC04). The ORFs and RdRp domains were represented by rectangular boxes. Positions of UGA (Trp) codons in each RdRp ORF are shown as black dots while UGG (Trp) codons are shown as green dots. The catalytic motif GDD in each deduced RdRp domain is also indicated. For each sequence, length of the RdRp domain in aa; and UGA and UGG codons in raw counts.


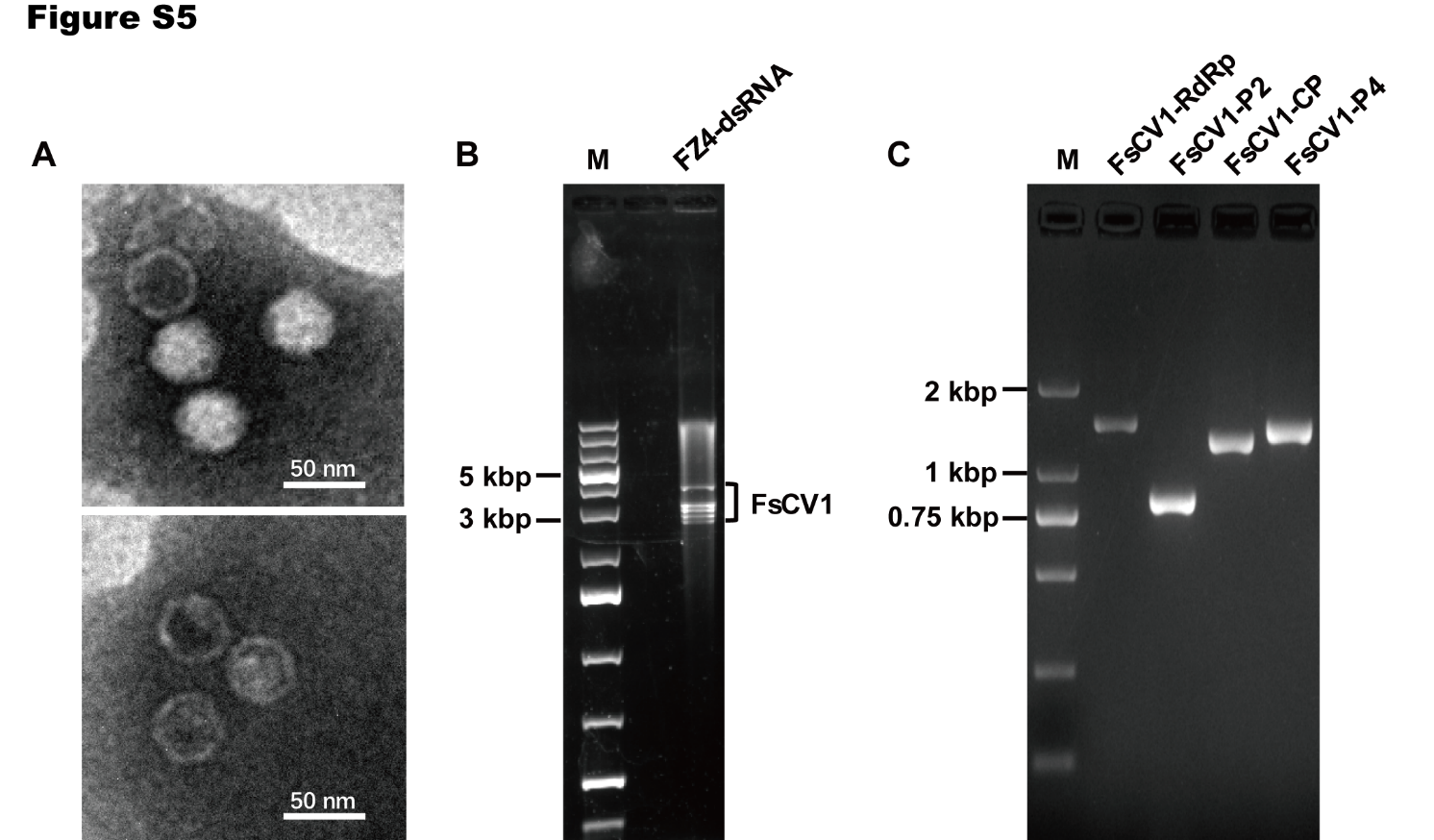


**Figure S5.** Puriﬁcation of virus-like particles from isolate FJ-FZ04. **(A)** Transmission electron micrograph of the phosphotungstic acid -stained virus particles. The scale bars indicate 50 nm. **(B)** Agarose gel electrophoresis of dsRNA extracted from virus particles. **(C)** RT-PCR detection of cDNA from dsRNA segment -1, -2, -3, and -4.
