# Supplementary material for: Virome Identification and Characterization of Fusarium sacchari and F. andiyazi: Causative Agents of Pokkah Boeng Disease in Sugarcane
Source: Front Microbiol. 2020 Feb 19;11:240. doi: 10.3389/fmicb.2020.00240 (PMC7042383; doi:10.3389/fmicb.2020.00240)
Supplement: Supplementary file 2 [file Data_Sheet_2.docx]

**Table S1.** *Fusarium sacchari* and *F. andiyazi* isolates from China used in this study.

| **Strain name** | ***Fusarium* species** | **HOST** | **collected from** | **DATE** | **Chinese province** |
| --- | --- | --- | --- | --- | --- |
| FJ-FZ04 | *Fusarium sacchari* | *Saccharum officinarum* | Sugarcane planting base in Fuzhou | 2012 | Fujian |
| FJ-FZ06 | *Fusarium sacchari* | *Saccharum officinarum* | Sugarcane planting base in Fuzhou | 2012 | Fujian |
| GX-FS01 | *Fusarium sacchari* | *Saccharum officinarum* | Sugarcane planting base in Fusui | 2017 | Guangxi |
| GX-FS03 | *Fusarium sacchari* | *Saccharum officinarum* | Sugarcane planting base in Fusui | 2017 | Guangxi |
| GX-FS09 | *Fusarium andiyazi* | *Saccharum officinarum* | Sugarcane planting base in Fusui | 2017 | Guangxi |
| GX-FS12 | *Fusarium sacchari* | *Saccharum officinarum* | Sugarcane planting base in Fusui | 2017 | Guangxi |
| GX-LC04 | *Fusarium sacchari* | *Saccharum officinarum* | Sugarcane planting base in Liucheng | 2012 | Guangxi |
| YN-DH06 | *Fusarium andiyazi* | *Saccharum officinarum* | Sugarcane planting base in Dehong | 2012 | Yunnan |
| YN-DH09 | *Fusarium andiyazi* | *Saccharum officinarum* | Sugarcane planting base in Dehong | 2012 | Yunnan |
| YN-BS34 | *Fusarium andiyazi* | *Saccharum officinarum* | Sugarcane planting base in Baoshan | 2013 | Yunnan |
| YN-BS38 | *Fusarium andiyazi* | *Saccharum officinarum* | Sugarcane planting base in Baoshan | 2013 | Yunnan |
| YN-SJ46 | *Fusarium andiyazi* | *Saccharum officinarum* | Sugarcane planting base in Shuangjiang | 2013 | Yunnan |
| YN-GM56 | *Fusarium andiyazi* | *Saccharum officinarum* | Sugarcane planting base in Gengma | 2013 | Yunnan |
| YN-GM64 | *Fusarium andiyazi* | *Saccharum officinarum* | Sugarcane planting base in Gengma | 2013 | Yunnan |
| HN-DZ | *Fusarium andiyazi* | *Saccharum officinarum* | Sugarcane planting base in Haikou | 2014 | Hainan |

**Table S2.** Primer pairs used to confirm viral sequences in strains of *Fusarium sacchari* and *F. andiyazi* isolates.

| **Name** | **Contig number** | **Primer name** | **Primer sequence** | **Amplicon size (bp)** | **Note** |
| --- | --- | --- | --- | --- | --- |
| FsCV1 RdRp | Contig 2971_seq1 | F | AGAACAGCAGCGTAGCGATCAC | 1,460 |  |
|  |  | R | GCAGCATACCCATCACCCATAAATC |  |  |
| FsCV1 P2 | Contig 18164_seq1 | F | GCAAGTGTACCGACTGCCGATAAG | 853 |  |
|  |  | R | ACTCGCTCATGAACGCCATCCT |  |  |
| FsCV1 CP | Contig 17958_seq1 | F | ACCGTTTGCTAAATAGCGTCACCAT | 1,309 |  |
|  |  | R | ACCCGCAACCCATGTACTACTGT |  |  |
| FsCV1 P4 | Contig 3153_seq1 | F | ACCATGACAACCACATACTGGAAGT | 1,457 |  |
|  |  | R | CCTGATCCTGGACCGTTAGCATT |  |  |
| FsHV1 | Contig 17922_seq1 | F | TCACGAGAGGTTGGACAG | 1,886 | Primers-designing region contains the defective RNA. |
| FsHV1-D RNA | Contig 17922_seq2 | R | TCTAGGCAGGTTGAACAGT | 206 |  |
| FaMV1-DH06 | Contig 10052_seq2 | F | CCTTTCCCTTAGCCAAACTCTCTCAC | 2,233 |  |
| FaMV1-DZ |  | R | GTTAGACGGTAGGACTGCGAGCTA |  |  |
| FsMV1-LC04 | Contig 17543_seq3 | F | AACTGGTTAGAATCCGTTCTCTATGGT | 2,383 |  |
| FaMV1- SJ46  FaMV1-BS38  FaMV1- GM64 |  | R | CGTAGATGCCAAAGATTCCCACATTT |  |  |
| FaMV2 | Contig 5918_seq1 | F | CTATACTAATGATATGATGATTGG | 1,066 |  |
|  |  | R | ATATGTCGTTTGTGACACATGAGT |  |  |
| FsALV1 | Contig 3638_seq1 | F | TGACCATTAGACAGCACGGCAC | 468 |  |
|  |  | R | GAAGTGGAGGGATTCTTGGCAAC |  |  |

**Table S3.** Viruses selected for phylogenetic analysis in this study

| **Family** | **Virus name** | **Virus protein for alignment** | **NCIB Accession number** |
| --- | --- | --- | --- |
| *Chrysoviridea* | ACDaCV | putative RNA-dependent RNA polymerase [Amasya cherry disease associated chrysovirus] | YP_001531163.1 |
|  | RSCV1 | putative RNA-dependent RNA polymerase [Raphanus sativas chrysovirus 1] | AFE83590.1 |
|  | BcCV1 | putative RNA-dependent RNA polymerase [Brassica campestris chrysovirus 1] | AKU48197.1 |
|  | CgCV1 | RNA-directed RNA-polymerase [Colletotrichum gloeosporioides chrysovirus 1] | ALW95408.1 |
|  | VdCV1 | RNA-dependent RNA polymerase [Verticillium dahliae chrysovirus 1] | YP_009507948.1 |
|  | CnCV1 | RNA-dependent RNA polymerase [Cryphonectria nitschkei chrysovirus 1] | YP_009507942.1 |
|  | AfCV1 | RNA-dependent RNA polymerase [Aspergillus fumigatus chrysovirus] | YP_009508104.1 |
|  | PcV | RNA-dependent RNA polymerase [Penicillium chrysogenum virus] | YP_392482.1 |
|  | AMaV | putative RNA-dependent RNA polymerase [Anthurium mosaic-associated virus] | ACU11563.1 |
|  | FoCV1 | putative RNA polymerase, partial [Fusarium oxysporum chrysovirus 1] | ABQ53134.1 |
|  | MpCV1 | RNA-dependent RNA polymerase [Macrophomina phaseolina chrysovirus 1] | ALD89090.1 |
|  | IjCV1 | RNA-dependent RNA polymerase [Isaria javanica chrysovirus 1] | YP_009337840.1 |
|  | PaCV | putative RNA-dependent RNA polymerase [Persea americana chrysovirus] | AJA37498.1 |
|  | FodV1 | RNA-dependent RNA polymerase [Fusarium oxysporum f. sp. dianthi mycovirus 1] | YP_009158913.1 |
|  | BdCV1 | putative RNA-dependent RNA polymerase [Botryosphaeria dothidea chrysovirus 1] | YP_009353026.1 |
|  | PjCV1 | 127 kDa protein [Penicillium janczewskii chrysovirus 1] | YP_009182332.1 |
|  | PjCV2 | ORF1 [Penicillium janczewskii chrysovirus 2] | ALO50149.1 |
|  | MoCV1-A | RNA dependent RNA polymerase [Magnaporthe oryzae chrysovirus 1] | YP_003858286.1 |
|  | MoCV1-B | RNA dependent RNA polymerase [Magnaporthe oryzae chrysovirus 1] | YP_008914864.1 |
|  | AbV1 | RNA-dependent RNA polymerase [Agaricus bisporus virus 1] | CAA64144.1 |
|  | HvV145S | RNA-dependent RNA polymerase [Helminthosporium victoriae 145S virus] | YP_052858.1 |
|  | BmCV1 | RNA-dependent RNA polymerase [Bipolaris maydis chrysovirus 1] | ARM36035.1 |
|  | GaCV1 | putative RNA-dependent RNA polymerase, partial [Grapevine associated chrysovirus-1] | ADO60926.1 |
|  | FgV-ch9 | RNA dependent RNA polymerase [Fusarium graminearum mycovirus-China 9] | ADU54123.1 |
|  | FgV2 | RNA-dependent RNA polymerase [Fusarium graminearum dsRNA mycovirus-2] | ADW08802.1 |
|  | TcV2 | RNA dependent RNA polymerase [Tolypocladium cylindrosporum virus 2] | CBY84993.1 |
|  | AmV1816 | putative RNA-dependent RNA polymerase, partial (endogenous virus) [Aspergillus mycovirus 1816] | ABX79996.1 |
| *Hypoviridae* | BHLV1 | polyprotein [Beihai hypo-like virus 1] | YP_009333297 |
|  | BSWV6 | polyprotein [Beihai sipunculid worm virus 6] | YP_009333562.1 |
|  | CHV3 | polyprotein [Cryphonectria hypovirus 3] | NP_051710.1 |
|  | CHV4 | polyprotein (endogenous virus) [Cryphonectria hypovirus 4] | YP_138519.1 |
|  | PlHV | polyprotein [Phomopsis longicolla hypovirus] | YP_009051683.1 |
|  | VcHV1 | unnamed protein product [Valsa ceratosperma hypovirus 1] | YP_005476604.1 |
|  | BcHV1 | polyprotein [Botrytis cinerea hypovirus 1] | YP_009480677.1 |
|  | SsHV1 | unnamed protein product [Sclerotinia sclerotiorum hypovirus 1] | YP_004782527.1 |
|  | SsHV1-A | polyprotein [Sclerotinia sclerotiorum hypovirus 1-A] | AWY10948.1 |
|  | SsHV2_5472 | polyprotein [Sclerotinia sclerotiorum hypovirus 2]/5472 | YP_008828161.1 |
|  | SsHV2_L | RNA-dependent RNA polymerase [Sclerotinia sclerotiorum hypovirus 2]/L | AHE13861.1 |
|  | SsHV2_SX247 | polyprotein [Sclerotinia sclerotiorum hypovirus 2]/SX247 | AIA61616.1 |
|  | SrHV1 | polyprotein [Sclerotium rolfsii hypovirus 1] | AZA15168.1 |
|  | SrHV2 | polyprotein [Sclerotium rolfsii hypovirus 2] | AZF86107.1 |
|  | SrHV3 | polyprotein [Sclerotium rolfsii hypovirus 3] | AZF86108.1 |
|  | SrHV4 | polyprotein [Sclerotium rolfsii hypovirus 4] | AZF86109.1 |
|  | SrHV5 | polyprotein [Sclerotium rolfsii hypovirus 5] | AZF86110.1 |
|  | SrHV7 | polyprotein [Sclerotium rolfsii hypovirus 7] | AZF86112.1 |
|  | SrHV8 | polyprotein [Sclerotium rolfsii hypovirus 8] | AZF86113.1 |
|  | CHV1-EP713 | hypothetical protein [Cryphonectria hypovirus 1] | NP_041091.1 |
|  | CHV2 | polyprotein [Cryphonectria hypovirus 2] | NP_613266.1 |
|  | AaHV1 | polyprotein [Alternaria alternata hypovirus 1] | QFR36339.1 |
|  | RsHV1 | polyprotein [Rhizoctonia solani hypovirus 1] | QDW92698.1 |
|  | MpHV1 | RNA-dependent RNA polymerase, partial [Macrophomina phaseolina hypovirus 1] | ALD89099.1 |
|  | WIV14 | polyprotein [Wuhan insect virus 14] | YP_009342443.1 |
|  | FgHV1 | hypothetical protein FgHV1gp2 [Fusarium graminearum hypovirus 1] | YP_009011065.1 |
|  | FgHV2 | polyprotein [Fusarium graminearum hypovirus 2] | YP_009130646.1 |
|  | RnHV1 | polyprotein [Rosellinia necatrix hypovirus 1] | YP_009448196.1 |
|  | RnHV2 | polyprotein [Rosellinia necatrix hypovirus 2] | BBB86776.1 |
|  | FlHV1 | polyprotein [Fusarium langsethiae hypovirus 1] | YP_009330037.1 |
|  | FpHV1 | polyprotein [Fusarium poae hypovirus 1] | BAV56305.1 |
| Fusariviruses | SrHV6 | polyprotein [Sclerotinia sclerotiorum hypovirus 6] | AZF86111.1 |
|  | AbV11 | replicase [Agaricus bisporus virus 11] | AQM49938.1 |
|  | AbFV1 | polyprotein [Alternaria brassicicola fusarivirus 1] | ALW95411.1 |
|  | RnFV1 | RNA dependent RNA polymerase [Rosellinia necatrix fusarivirus 1] | BAP16392.1 |
|  | PtFV1 | 175 kDa protein [Pleospora typhicola fusarivirus 1] | ALO50136.1 |
|  | FpFV1 | RNA-dependent RNA polymerase [Fusarium poae fusarivirus 1] | BAV56303.1 |
|  | MpRV1 | RNA-dependent RNA polymerase [Macrophomina phaseolina single-stranded RNA virus 1] | ALD89094.1 |
|  | FgV1 | RNA-dependent RNA polymerase [Fusarium graminearum dsRNA mycovirus-1] | AAT07067.2 |
|  | PrRV1 | RNA-dependent RNA polymerase [Penicillium roqueforti ssRNA mycovirus 1] | AII99895.1 |
| Narnaviridae | FcMV2-1 | RNA-dependent RNA polymerase [Fusarium circinatum mitovirus 2-1] | AHI43534.1 |
|  | FcMV2-2 | RNA-dependent RNA polymerase, partial [Fusarium circinatum mitovirus 2-2] | AHI43535.1 |
|  | FpMV1 | RNA-dependent RNA polymerase [Fusarium poae mitovirus 1] | YP_009272898.1 |
|  | AbMV | RNA-dependent RNA polymerases [Alternaria brassicicola mitovirus] | AKN79252.1 |
|  | NoMV2 | RNA-dependent RNA polymerase [Nigrospora oryzae mitovirus 2] | AZP53929.1 |
|  | GaMRV-S2 | putative RNA-dependent RNA polymerase [Gremmeniella abietina mitochondrial RNA virus S2] | YP_077184.1 |
|  | OnuMV4 | RNA-dependent RNA polymerase, putative [Ophiostoma mitovirus 4] | NP_660179.1 |
|  | SsMV4 | RNA-dependent RNA polymerases [Sclerotinia sclerotiorum mitovirus 4] | AMT92141.1 |
|  | FpMV2 | RNA-dependent RNA polymerase [Fusarium poae mitovirus 2] | YP_009272899.1 |
|  | FcoMV1 | RNA-dependent RNA polymerase [Fusarium coeruleum mitovirus 1] | YP_009126873.1 |
|  | FcMV1 | RNA-dependent RNA polymerase [Fusarium circinatum mitovirus 1] | AHI43533.1 |
|  | FgMV1 | RNA-dependent RNA polymerase [Fusarium globosum mitovirus 1] | YP_009126872.1 |
|  | OnuMV5 | RNA-dependent RNA polymerase, putative [Ophiostoma mitovirus 5] | NP_660180.1 |
|  | SsMV1 | RNA-dependent RNA polymerases [Sclerotinia sclerotiorum mitovirus 1] | AEX91878.1 |
|  | OnuMV6 | RNA-dependent RNA polymerase, putative [Ophiostoma mitovirus 6] | NP_660181.1 |
|  | SsMV8 | RNA-dependent RNA polymerases [Sclerotinia sclerotiorum mitovirus 8] | AHF48624.1 |
|  | SsMV15 | RNA-dependent RNA polymerase [Sclerotinia sclerotiorum mitovirus 15] | AHF48631.1 |
|  | SsMV17 | RNA-dependent RNA polymerase [Sclerotinia sclerotiorum mitovirus 17] | ALD89134.1 |
|  | SsMV19 | RNA-dependent RNA polymerase [Sclerotinia sclerotiorum mitovirus 19] | ALD89136.1 |
|  | SsMV20 | RNA-dependent RNA polymerase [Sclerotinia sclerotiorum mitovirus 20] | ALD89137.1 |
|  | SsMV7-A2 | RNA-dependent RNA polymerases [Sclerotinia sclerotiorum mitovirus 7-A2] | AWY10970.1 |
|  | RsMV2 | RNA-dependent RNA polymerase [Rhizoctonia solani mitovirus 2] | ALD89121.1 |
|  | RsMV11 | RNA-dependent RNA polymerase [Rhizoctonia solani mitovirus 11] | ALD89116.1 |
|  | OnuMV1a | RNA-dependent RNA polymerase [Ophiostoma mitovirus 1a] | CAJ32466.1 |
|  | SsMV27 | RNA-dependent RNA polymerase [Sclerotinia sclerotiorum mitovirus 27] | AWY10985.1 |
|  | FbMV1-Ep-BL13 | RNA dependent RNA polymerase [Fusarium boothii mitovirus 1] | BBG56022.1 |
|  | FbMV1-Ep-BL14 | RNA dependent RNA polymerase [Fusarium boothii mitovirus 1] | BBG56023.1 |
|  | FbMV1-Ep-28 | RNA dependent RNA polymerase [Fusarium boothii mitovirus 1] | BBG56024.1 |
|  | FpMV3 | RNA-dependent RNA polymerase [Fusarium poae mitovirus 3] | YP_009272900.1 |
|  | SsMV28 | RNA-dependent RNA polymerase [Sclerotinia sclerotiorum mitovirus 28] | AWY10986.1 |
|  | BcMV1 | RNA-dependent RNA polymerase [Botrytis cinerea mitovirus 1] | YP_002284334.2 |
|  | FpMV4 | RNA-dependent RNA polymerase [Fusarium poae mitovirus 4] | YP_009272901.1 |
|  | OnuMV3a | RNA-dependent RNA polymerase [Ophiostoma mitovirus 3a] | NP_660176.1 |
|  | SsMV3 | RNA-dependent RNA polymerases [Sclerotinia sclerotiorum mitovirus 3] | YP_009182164.1 |
|  | SsMV30 | RNA-dependent RNA polymerase [Sclerotinia sclerotiorum mitovirus 30] | AWY10988.1 |
|  | BcMV2 | RNA dependent RNA polymerase [Botrytis cinerea mitovirus 2] | CEZ26301.1 |
|  | SsMV18 | RNA-dependent RNA polymerase [Sclerotinia sclerotiorum mitovirus 18] | ALD89135.1 |
|  | BcMV4 | RNA dependent RNA polymerase [Botrytis cinerea mitovirus 4] | YP_009182163.1 |
|  | EmMV6 | RNA-dependent RNA polymerase [Entomophthora muscae mitovirus 6] | QCF24465.1 |
|  | RsMV6 | RNA-dependent RNA polymerase [Rhizoctonia solani mitovirus 6] | ALD89125.1 |
|  | EmMV7 | RNA-dependent RNA polymerase [Entomophthora muscae mitovirus 7] | QCF24466.1 |
|  | EmMV2 | RNA-dependent RNA polymerase [Entomophthora muscae mitovirus 2] | QCF24461.1 |
|  | EmMV1 | RNA-dependent RNA polymerase [Entomophthora muscae mitovirus 1] | QCF24460.1 |
|  | CrMV2 | RNA-dependent RNA polymerase [Cronartium ribicola mitovirus 2] | YP_009259481.1 |
|  | CcMV1b | RNA-dependent RNA polymerase [Cryphonectria cubensis mitovirus 1b] | AAR01971.1 |
|  | SsMV2 | RNA-dependent RNA polymerases [Sclerotinia sclerotiorum mitovirus 2] | YP_009551566.1 |
|  | CrMV5 | RNA-dependent RNA polymerase [Cronartium ribicola mitovirus 5] | YP_009259487.1 |
|  | CrMV4 | RNA-dependent RNA polymerase [Cronartium ribicola mitovirus 4] | YP_009259483.1 |
|  | GmMV1 | RNA-dependent RNA polymerase [Gigaspora margarita mitovirus 1] | YP_009553175.1 |
|  | AzfiMV1 | TPA_inf: RNA-dependent RNA polymerase [Azolla filiculoides mitovirus 1] | DAB41741.1 |
|  | DapiMV1 | TPA_inf: RNA-dependent RNA polymerase [Dahlia pinnata mitovirus 1] | DAB41747.1 |
|  | AmarMV1 | TPA_inf: RNA-dependent RNA polymerase [Ambrosia artemisiifolia mitovirus 1] | DAB41740.1 |
|  | BevuMV1 | TPA_inf: RNA-dependent RNA polymerase [Beta vulgaris mitovirus 1] | DAB41757.1 |
|  | CasaMV1 | TPA_inf: RNA-dependent RNA polymerase [Cannabis sativa mitovirus 1] | DAB41746.2 |
|  | SochMV1 | TPA_inf: RNA-dependent RNA polymerase [Solanum chacoense mitovirus 1] | DAB41743.1 |
|  | OxruMV1 | TPA_inf: RNA-dependent RNA polymerase [Oxybasis rubra mitovirus 1] | DAB41745.1 |
|  | CpMV1 | putative RNA-dependent RNA polymerase [Cryphonectria parasitica mitovirus 1-NB631] | AAA61703.1 |
|  | RsMV34 | putative RNA-dependent RNA polymerase [Rhizoctonia solani mitovirus 34] | QDW65424.1 |
|  | MpMV2 | RNA-dependent RNA polymerase, partial [Macrophomina phaseolina mitovirus 2] | ALD89101.1 |
|  | RsMV8 | RNA-dependent RNA polymerase [Rhizoctonia solani mitovirus 8] | ALD89127.1 |
|  | RsMV27 | putative RNA-dependent RNA polymerase [Rhizoctonia solani mitovirus 27] | QDW65417.1 |
|  | RsMV30 | putative RNA-dependent RNA polymerase [Rhizoctonia solani mitovirus 30] | QDW65420.1 |
|  | RsMV29 | putative RNA-dependent RNA polymerase [Rhizoctonia solani mitovirus 29] | QDW65419.1 |
|  | RsMV38 | putative RNA-dependent RNA polymerase [Rhizoctonia solani mitovirus 38] | QDW65426.1 |
|  | RsMV21 | putative RNA-dependent RNA polymerase [Rhizoctonia solani mitovirus 21] | QDW65413.1 |
|  | MpMV3 | putative replicase [Macrophomina phaseolina mitovirus 3] | AMM45292.1 |
|  | RsMV25 | putative RNA-dependent RNA polymerase [Rhizoctonia solani mitovirus 25] | QDW65415.1 |
|  | RsMV37 | putative RNA-dependent RNA polymerase [Rhizoctonia solani mitovirus 37] | QDW65425.1 |
|  | MpMV1 | RNA-dependent RNA polymerase [Macrophomina phaseolina mitovirus 1] | ALD89100.1 |
|  | GmMV2 | RNA-dependent RNA polymerase [Gigaspora margarita mitovirus 2] | YP_009553587.1 |
|  | GmMV3 | RNA-dependent RNA polymerase [Gigaspora margarita mitovirus 3] | YP_009551960.1 |
|  | OnuMV7 | RNA-dependent RNA polymerase [Ophiostoma mitovirus 7] | AGT55877.1 |
|  | ScnV20S | RNA-dependent RNA polymerase [Saccharomyces 20S RNA narnavirus] | NP_660178.1 |
|  | ScnV23S | RNA-dependent RNA polymerase [Saccharomyces 23S RNA narnavirus] | NP_660177.1 |
|  | RnnV23S | RNA-dependent RNA polymerase, partial [Rhizopus microsporus 23S narnavirus] | QBC65281.1 |
| Ourmia-like mycovirus | SsOLV2 | RNA-dependent RNA polymerase, partial [Sclerotinia sclerotiorum ourmia-like virus 2] | ALD89139.1 |
|  | BoOLV | RNA dependent RNA polymerase [Botrytis ourmia-like virus] | CEZ26310.1 |
|  | RsOLV1 | RNA-dependent RNA polymerase, partial [Rhizoctonia solani ourmia-like virus 1] | ALD89131.1 |
| Ourmia-like invertebrate virus | BhNLV1 | Beihai narna-like virus 1 strain BWBFG61141 RNA-dependent RNA polymerase gene, complete cds. | KX883515.1 |
|  | WzNLV3 | RNA-dependent RNA polymerase [Wenzhou narna-like virus 3] | YP_009336520.1 |
|  | HbNLV3 | RNA-dependent RNA polymerase [Hubei narna-like virus 3] | YP_009337787.1 |
| Ourmiavirus | CsVC | putative RNA dependent RNA polymerase [Cassava virus C] | YP_003104770.1 |
|  | EpCV | Epirus cherry virus segment RNA1 putative RNA dependent RNA polymerase mRNA, complete cds | EU770620.1 |
|  | OuMV | putative RNA dependent RNA polymerase [Ourmia melon virus] | YP_002019757.1 |
| *Hepeviridae* | swHEV | nonstructural protein [Swine hepatitis E virus] | AHZ44444.1 |
|  | avHEV | non-structural polyprotein [Orthohepevirus B] | AAS45830.1 |
|  | HEV_sp. | non-structural polyprotein [Hepevirus sp.] | ATY47660.1 |
|  | fHEV | polyprotein [Ferret hepatitis E virus] | BAT70058.1 |
|  | HEV | polyprotein [Hepatitis E virus] | NP_056779.1 |
| *Benyviridae* | HBLV1 | hypothetical protein [Hubei Beny-like virus 1] | APG77690.1 |
|  | RSNV | replication-associated protein [Rice stripe necrosis virus] | ABU94739.2 |
|  | MILV | RNA-dependent RNA polymerase [Mangifera indica latent virus] | AMQ23297.1 |
|  | BSBMV | polyprotein [Beet soil-borne mosaic virus] | NP_612601.1 |
|  | BdMoV | 249 kDa protein [Burdock mottle virus] | YP_008219063.1 |
|  | BNYVV | RNA-dependent RNA polymerase [Beet necrotic yellow vein virus] | NP_705701.1 |
| *Togaviridae* | SINV | RNA-dependent RNA polymerase [Sindbis virus] | QGA70907.1 |
|  | VEEV | RNA polymerase nsP4 [Everglades virus] | YP_009509017.1 |
| Alphavirus-like | RsALV1 | RNA-dependent RNA polymerase, partial [Rhizoctonia solani alphavirus-like virus 1] | QDW81323.1 |
|  | RsALV2 | RNA-dependent RNA polymerase, partial [Rhizoctonia solani alphavirus-like 2] | QDW81322.1 |
|  | RsALV3 | RNA-dependent RNA polymerase [Rhizoctonia solani alphavirus-like 3] | QDW81316.1 |
|  | SsRV-L | polyprotein [Sclerotinia sclerotiorum RNA virus L] | ACE88957.1 |
|  | SraLV1 | polyprotein [Sclerotium rolfsii alphavirus-like virus 1] | AZF86093.1 |
|  | SraLV2 | polyprotein [Sclerotium rolfsii alphavirus-like virus 2] | AZF86094.1 |
|  | SraLV3 | polyprotein [Sclerotium rolfsii alphavirus-like virus 3] | AZF86095.1 |
|  | MiRV1 | polyprotein [Morchella importuna RNA virus 1] | AZT88619.1 |
